# Multiscale Computational Dissection of CCRL2-Mediated Chemerin Presentation

**DOI:** 10.1021/acs.jcim.5c01871

**Published:** 2025-12-11

**Authors:** Arianna Migliorini, Samuele Di Cristofano, Klevia Dishnica, Alessandro Marchetto, Rui Pedro Ribeiro, Mattia Laffranchi, Elena Cerioni, Francesco Quilli, Eleonora Bonanni, Alejandro Giorgetti, Giulia Rossetti, Silvano Sozzani, Tiziana Borsello, Domenico Raimondo

**Affiliations:** † Department of Molecular Medicine, Laboratory Affiliated to Istituto Pasteur Italia - Fondazione Cenci Bolognetti, 9311 Sapienza University of Rome, Viale Regina Elena 291, Rome 00161, Italy; ‡ Department of Chemistry, Bioscience and Environmental Engineering, Faculty of Science and Technology, University of Stavanger, Kristine Bonnevies vei 22, Stavanger 4021, Norway; § Department of Biotechnology, 19051 University of Verona, Strada le Grazie 15, Verona 37134, Italy; ∥ Computational Biomedicine, 28334 Forschungszentrum Jülich, Wilhelm-Johnen-Straße, Jülich 52428, Germany; ⊥ Department of Biology, Faculty of Mathematics, Computer Science and Natural Sciences, RWTH Aachen University, Templergraben 59, Aachen D-52062, Germany; # Department of Pharmacological and Biomolecular Sciences, University of Milan, Via Balzaretti 9, Milan 20133, Italy; ¶ Department of Neuroscience, Mario Negri Institute of Pharmacological Research IRCCS, Via Mario Negri 2, Milan 20156, Italy; 8 Jülich Supercomputing Center, 28334 Forschungszentrum Jülich, Wilhelm-Johnen-Straße, Jülich 52428, Germany; 9 Department of Neurology, University Hospital Aachen, RWTH Aachen University, Pauwelsstraße 30, Aachen 52074, Germany

## Abstract

Chemokine-like receptor CCRL2 is a nonsignaling atypical GPCR that presents chemerin to its cognate receptor CMKLR1 (ChemerinR1), a process essential for the recruitment of inflammatory cells. Despite their biological importance, the structural determinants of CCRL2–chemerin recognition remain poorly defined. Here, we present a comprehensive multiscale computational study that integrates coarse-grained and all-atom molecular dynamics simulations with structural modeling to investigate CCRL2–chemerin interaction. Our results reveal a flexible yet stable binding interface primarily mediated by chemerin’s β1 strand and CCRL2’s extracellular loop 2, while the C-terminal region of chemerin remains accessible for CMKLR1 engagement. Electrostatic interactions between CCRL2 N-terminus and chemerin’s loop 3 further stabilize the complex without triggering intracellular signaling. A modeled ternary CCRL2–chemerin–CMKLR1 complex provides a putative mechanistic framework in which CCRL2 aligns chemerin to promote efficient CMKLR1 activation. Mapping of naturally occurring missense variants onto this interface suggests that sequence variation at specific residues may influence receptor–ligand stability and function. Together, these findings suggest a structural basis of CCRL2-mediated chemerin presentation and may help improve our understanding of its role in immune signaling.

## Graphical abstract

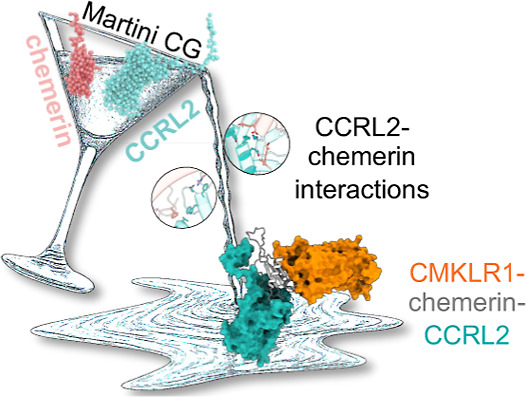

## Introduction

CCRL2 is a class A G-protein-coupled receptor (GPCR) structurally related to the atypical chemokine receptor (ACKRs) family and shares several of its features.^1^ CCRL2 is considered a nonsignaling receptor because alterations in the conserved “DRYLAIV” motif^1^ prevent G-protein coupling upon ligand binding. Moreover, CCRL2 does not undergo rapid internalization, does not promote ligand scavenging,^2,3^ and does not activate β-arrestin-dependent signaling.^2–5^ To date, the only experimentally validated ligand for CCRL2 is chemerin,^4^ a nonchemokine chemotactic protein.^2,3,6,7^

Although CCRL2 does not directly promote cell migration, it plays a key role in leukocyte recruitment under inflammatory conditions through two main mechanisms.^8^ First, CCRL2 concentrates chemerin on the surface of CCRL2-expressing cells, facilitating β1-integrin-dependent arrest and adhesion of circulating CMKLR1-positive leukocytes.^8–10^ Second, it modulates neutrophil migration by forming CCRL2/CXCR2 heterodimers in mouse neutrophils.^8^ Chemerin is secreted as an inactive precursor of 143 amino acids (prochemerin).^11^ Serine proteases from the coagulation, fibrinolytic, and inflammatory cascades process its C-terminus to generate active chemerin, which can subsequently be inactivated by further C-terminal cleavage.^4^ While chemerin binding to CCRL2 does not trigger intracellular signaling, it activates two other GPCRs, namely CMKLR1 (Chemerin_1_, the functional chemerin receptor) and GPR1 (Chemerin_2_, the putative chemerin scavenger receptor).^12,13^

It has been proposed that CCRL2 acts as a chemerin-presenting receptor by binding chemerin through its N-terminal domain while leaving the C-terminal sequence exposed for CMKLR1 engagement.^2^ Extensive structural and experimental studies on CMKLR1 support this model.^5,11,14–17^ More recently, experimental studies,^12^ corroborated by cryo-EM data, demonstrated that full activation of CMKLR1 requires not only chemerin’s C-terminal domain but also residues within its core, providing a detailed characterization of this interaction.^18^ In contrast, the CCRL2–chemerin interface remains poorly characterized, with no structural or functional studies elucidating its binding mechanism or its role within the CCRL2–chemerin–CMKLR1 axis. What is known so far is that CCRL2 binds specifically only to chemerin,^7^ and its binding affinity for its ligand is the lowest among the three chemerin receptors.^6^

To address this gap, we employed a multiscale computational approach to define the binding mode of chemerin to CCRL2. Using a hierarchical workflow, we combined coarse-grained molecular dynamics (CG-MD) and all-atom molecular dynamics (AA-MD) simulations to dissect the mechanisms of CCRL2–chemerin recognition (Scheme 1).

**Scheme 1:**
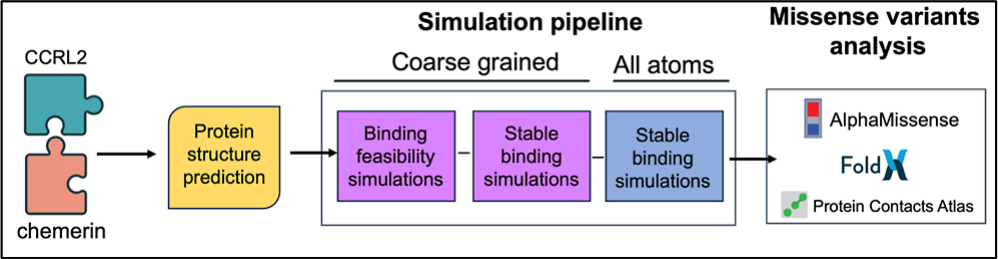
Multiscale Computational Workflow for Characterizing CCRL2–chemerin Interactions^a^

a Schematic representation of the integrated computational pipeline used to investigate the binding mechanism between CCRL2 and chemerin. The workflow begins with the protein structure prediction for CCRL2 and chemerin using AlphaFold. This is followed by three stages of molecular dynamics (MD) simulations: (1) *Binding feasibility simulations* using coarse-grained (CG) models to assess the spontaneous chemerin binding to CCRL2; (2) *Stable binding simulations* using CG-MD to refine stable conformations of the complex and identify key interaction sites; (3) *Stable binding simulations* at all-atom resolution to characterize atomic-level contacts and dynamics of the CCRL2–chemerin interface. The final stage involves pathogenicity prediction and mutational impact analysis using AlphaMissense and FoldX, integrating simulation-derived contact maps to assess the structural effects of naturally occurring variants at the CCRL2–chemerin–CMKLR1 interface.

We first performed a series of CG-MD simulations, initializing CCRL2 in an extended N-terminal conformation and positioning the N-terminal region of chemerin approximately 30 Å away. To explore spontaneous protein–protein association, we carried out 26 independent simulation replicas, yielding an aggregate of approximately 80 μs. Representative bound conformations from 11 of these trajectories were then subjected to additional CG-MD simulations to refine the binding pose and identify initial contact regions. Finally, we performed AA-MD simulations on the most representative coarse-grained stable binding conformations to obtain atomic-level insights and identify key interacting residues. In addition, we mapped naturally occurring missense variants onto the modeled interface to evaluate whether sequence variation could influence CCRL2–chemerin–CMKLR1 interactions. Overall, this study provides new mechanistic insights into how CCRL2 could present chemerin on the cell surface and facilitate localized chemotactic gradients that guide the recruitment of CMKLR1-positive leukocytes.

## Methods

### 3D Modeling of CCRL2 and Chemerin

The full-length CCRL2 isoform B sequence (residues 1–344; UniProt ID: O00421) was used without N- or C-terminal truncations to model both active and inactive conformations via a modified AlphaFold v2.0.1.^19^ For downstream MD, we selected the inactive-like CCRL2 model truncated at residue 318, and used the bioactive chemerin fragment (residues 21–137). To date, no experimentally determined structure of CCRL2 is available. Although CCRL2 does not trigger intracellular signaling pathways, we modeled both its active- and inactive-like conformational states using templates from signaling-competent chemokine receptors.^3^

This approach was taken due to the lack of definitive structural evidence explaining CCRL2’s inability to signal. While CCRL2 is known to lack the canonical DRYLAIV motif required for G protein activation,^2,8^ it remains unclear whether the receptor can adopt an active-like conformation upon chemerin binding. Thus, the possibility that CCRL2 assumes an active-like structure similar to other chemokine receptors yet fails to initiate signaling due to the absence of the DRYLAIV motif cannot be excluded.^2^ Conversely, it is also plausible that CCRL2 adopts an inactive-like conformation in the absence of a ligand.

For this reason, CCRL2 was modeled in two conformations: active and inactive. We chose to perform MD simulations using the inactive form of CCRL2, as this conformation corresponds to the unbound state of the receptor, and our goal was to explore the binding process starting from a chemerin-unbound configuration. Since isoform B is reported to be overexpressed under proinflammatory conditions,^8^ this isoform was selected for modeling. Structure prediction was performed using a modified version of AlphaFold v2.0.1.^19^ The modeling approach guided the modified version of AlphaFold to sample alternative conformations of topologically diverse transporters and GPCRs, incorporating CKR (chemokine receptor) family templates.^20^ Template selection was carried out using HHpred, which identified homologous proteins based on sequence similarity.^21^ For active-state modeling, four templates were selected: US28 (PDB: 4XT1), CXCR2 (PDB: 6LFO), CXCR4 (PDB: 4RWS, 2K04). For inactive-state modeling, templates included CXCR4 (PDB: 3ODU), CCR5 (PDB: 5UIW), CCR2 (PDB: 6GPX), and AT1 (PDB: 4ZUD). A total of 50 active and 50 inactive models were generated. Since no experimental structure was available as a reference, to select representative models, we used template modeling (TM) score,^22,23^ a metric ranging from 0 to 1, that quantifies the structural similarity between two protein backbones (Cα atoms), with higher values indicating greater similarity (Supporting Information Figure S1A).^22,23^ We selected the active and inactive models that showed a TM score of 0.77. As illustrated in Supporting Information Figure S1B, alignment of the Cα atoms of the transmembrane helices reveals that the active-like model exhibits an outward displacement of helices VI and VII, while the inactive model adopts a more inward-facing configuration. This conformational difference is consistent with the global toggle switch model, commonly observed in seven-transmembrane receptors. This model describes activation as a vertical seesaw motion of TM-VI and, to a lesser extent, TM-VII, around pivot points defined by highly conserved proline residues, which are also present in CCRL2.^24^ The inactive model was selected as the starting structure for the MD simulations. This choice is consistent with previous studies,^25,26^ where the inactive conformation is typically used as a starting point to simulate apo states.

For chemerin, we employed AlphaFold v2.0.1 ^19^ to predict the protein’s full-length conformation. For subsequent MD simulation, we focused on the bioactive form spanning residues 21 to 137. This form excludes the signal peptide at the N-terminal and the six amino acids at the C-terminal domain necessary for chemerin activation.

To evaluate the structural accuracy of the AlphaFold2-predicted chemerin model used in our simulations, which was generated prior to the release of recent cryo-EM data, we compared it to two newly resolved chemerin structures: PDB code 8ZJG^27^ and PDB code 8XGM.^18^ As the most recent structure, the protein with PDB code 8ZJG, released on January 22, 2025, in complex with CMKLR1, lacks several regions of chemerin, structural comparison was performed using the 8XGM structure, where chemerin was resolved in complex with GPR1 (Supporting Information Figure S1B). Excluding the N-terminal and C-terminal domains from the analysis (residues 21–23 and 140–257), the Cα RMSD between the predicted model and the experimental structure was only 0.01 nm, indicating a negligible conformational deviation. These findings confirm that the modeled chemerin structure reliably reflects the native folding and spatial organization observed experimentally, thereby supporting its suitability for our subsequent MD simulations.

### CCRL2 to Chemerin Coarse-Grained Binding Feasibility MD Simulations

In the coarse-grained binding feasibility simulations, chemerin was placed >30 Å from CCRL2’s extracellular N-terminal domain to avoid placement bias (Supporting Information Figure S2A). To determine the spatial arrangement of membrane proteins relative to the hydrocarbon core of the lipid bilayer, we used the Orientation of Proteins in Membranes (OPM) database.^28^ The PDB structure obtained from OPM was then converted into a coarse-grained representation using the martinize2 Python script.^29^ The coarse-grained CCRL2 model was subsequently embedded into a symmetric 1-palmitoyl-2-oleoyl-*sn*-glycero-3-phosphocholine (POPC) bilayer using the INSANE (INSert membrANE) protocol, ensuring proper matching of the hydrophobic thickness and transmembrane regions of CCRL2.^30^ We constructed a rectangular simulation box of 15 × 15 × 27 nm^3^, maintaining a 2.5 nm separation between periodic images in the XY plane, and solvated the system with coarse-grained water molecules. Finally, system neutrality was ensured by adding 0.15 M NaCl using the GROMACS tool gmx genion. The system contained a total of 51682 atoms: 8125 POPC, 555 Cl^–^, 549 Na^+^, 41336 water molecules, and 1118 protein atoms.

The MARTINI3 force field parameters^31^ were employed, along with elastic network restraints applied to the backbone beads with a force constant of 700 kJ/mol/nm^2^ and cutoff distances between 0.5 and 0.9 nm to maintain the protein secondary and tertiary structures.^32^

Following system preparation, energy minimization (EM) was performed to remove steric clashes and optimize the initial structure. The system was minimized using the steepest descent algorithm, as implemented in GROMACS 2020.5,^33^ with the following parameters: harmonic position restraints of 1000 kJ/mol/nm^2^ were applied to the backbone (BB) atoms of both CCRL2 and chemerin, the maximum force convergence criterion was set to 1000 kJ/mol/nm, with an energy step size of 0.01 nm. A maximum of 500,000 steps was allowed for the minimization process. To handle nonbonded interactions, a neighbor list update frequency of 1 step was used, with the list determined via a grid-based method. Electrostatic interactions were treated using particle mesh Ewald (PME)^34^ with a cutoff of 1.1 nm for short-range interactions. van der Waals (vdW) interactions were truncated to 1.1 nm. Periodic boundary conditions (PBCs) were applied in all directions (*xyz*).

After EM, we proceeded with equilibrating the solvent and ions around the proteins.

The system underwent equilibration in two stages: canonical (NVT) ensemble followed by isothermal–isobaric (NPT) ensemble, ensuring a well-equilibrated bilayer-protein environment before production MD simulations. NVT equilibration was performed for 10 ns using a velocity-rescaling (v-rescale) thermostat^35^ with separate temperature coupling for protein, POPC, and solvent, maintaining each at 303 K with a coupling constant of 1.0 ps. Nonbonded interactions were handled using reaction-field electrostatics with ε_r_ = 15 and a cutoff of 1.1 nm. vdW interactions were truncated at 1.1 nm by using a potential-shift-Verlet modifier, and neighbor lists were updated every 20 steps by using the Verlet cutoff scheme. Initial velocities were generated from a Maxwell distribution at 303 K. Following NVT equilibration, the system was equilibrated for an additional 10 ns under semi-isotropic pressure coupling to allow bilayer relaxation. The Berendsen barostat was applied with a time constant of 4.0 ps, maintaining pressure at 1.0 bar in both lateral (*xy*) and normal (*z*) directions with a compressibility of 4.5 × 10^–5^ bar^–1^.

Production runs were performed using a positional restraint of 200 kJ·mol^–1^·nm^–2^ on the last part of the N-terminal domain (residues 20–31) to maintain its structure and prevent aggregation in the CG-MD simulation. A time step of 20 fs was used. Temperature was controlled at 303 K using the v-rescale thermostat with a coupling constant of 1.0 ps, applied separately to protein, POPC, and solvent. The semi-isotropic Parrinello–Rahman pressure coupling was applied to maintain a pressure of 1 bar. Equilibration and production run were performed using GROMACS 2020.5.^33^ Finally, 26 independent simulations were performed, each initialized with different atomic velocities. The choice of running this large ensemble of MD simulations was driven by the need to obtain preliminary evidence of chemerin binding to CCRL2, given the lack of experimental structural data and uncertainty about whether binding would occur at all within accessible time scales. In this first stage, the aim was to *quantitatively* explore the configurational space of chemerin around CCRL2 to identify potential bound conformations rather than characterize the binding pathway. Notably, the stable contacts identified through this broad exploration were later confirmed to persist in subsequent atomistic simulations. Each of the 26 simulations was initially run for 1.5 μs, after which trajectories were visually and quantitatively inspected to assess whether chemerin approached CCRL2. To balance computational efficiency with biological relevance, simulations in which chemerin remained far from CCRL2 were not extended further (Supporting Information Figures S2B,D). By contrast, simulations in which chemerin approached CCRL2 were extended to 3 μs (Supporting Information Figure S2C,E), and those showing either clear evidence or a consistent trend toward binding were further extended to 4.5 μs (Figure 1C). With this adaptive protocol, we defined 11 stable binding trajectories (4.5 μs), 4 intermediate trajectories (3 μs), and 11 unbound trajectories (1.5 μs). Figure 1A presents only the 11 productive binding simulations (hereafter, *CG-binding_feasibility_simulations*), whereas the complete results for all 26 replicas are provided in Supporting Information Table S1 and Figure S2B–E. This strategy enabled an assessment of chemerin binding feasibility while ensuring transparent reporting of all trajectories. For clarity, the 11 productive simulations (replicas 1, 4, 6, 7, 9, 10, 11, 13, 16, 22, and 26) are referred to as simulations 1–11 throughout the manuscript.

**Figure 1:**
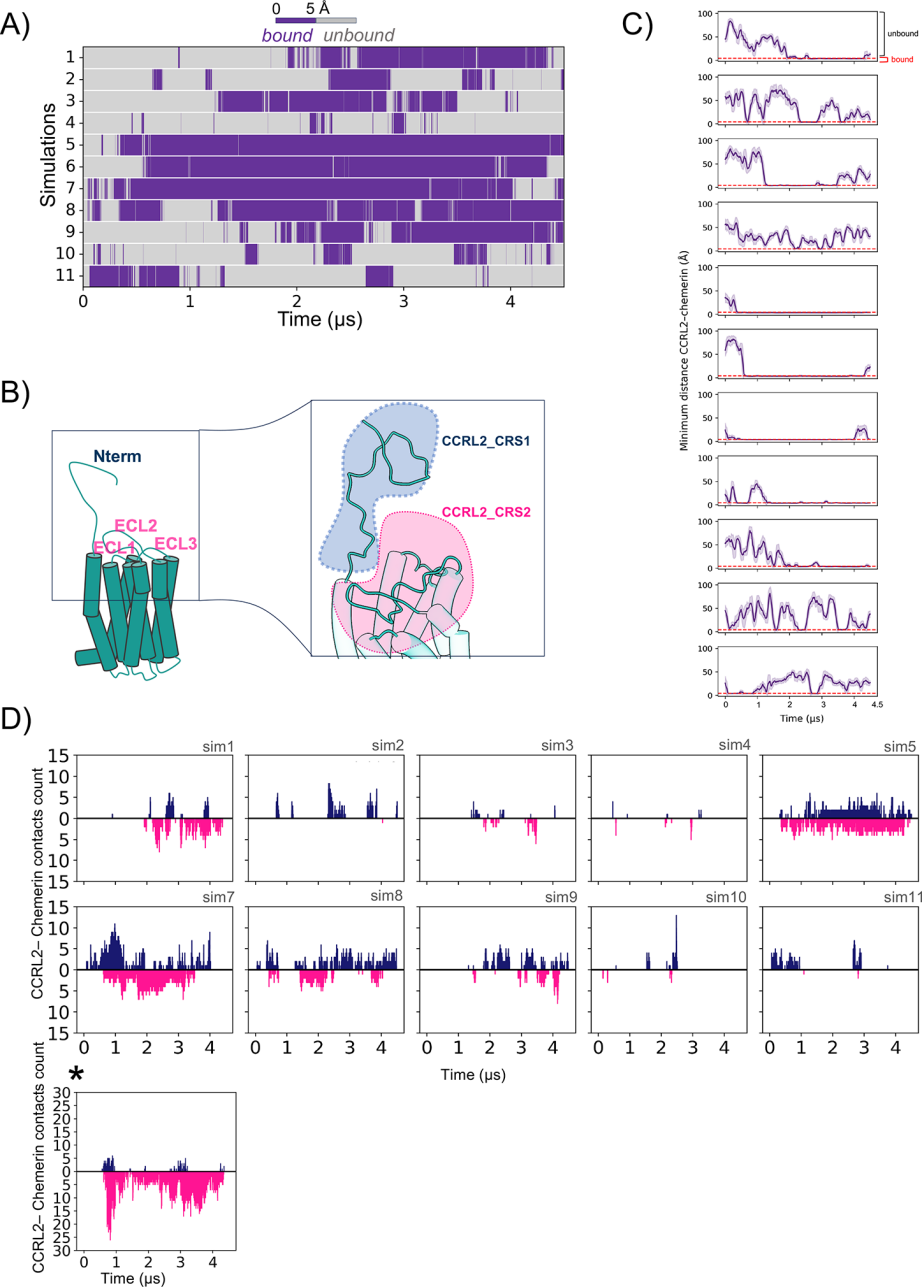
Two-step binding mechanism of CCRL2 and chemerin revealed by CG-MD simulations. (A) The minimum distance between CCRL2 and chemerin is plotted over 4.5 μs across the 11 selected *CG-binding_feasibility_simulations*. Each frame was color-coded based on the minimum distance between any atoms of the two proteins: gray regions represent the unbound regime (distance >5 Å), while purple regions represent the ligand-bound regime (distance <5 Å). (B) Structural representation of the two-site interaction. CCRL2 is shown in light blue. The CRS1 (chemokine recognition site 1), involving contacts with the N-terminal region of CCRL2, is highlighted in blue, whereas the CRS2 (chemokine recognition site 2), involving extracellular loops 1 (ECL1), 2 (ECL2), and 3 (ECL3) with the ECL2 of CCRL2, is highlighted in magenta. (C) Minimum distance between any atom of CCRL2 and any atom of chemerin across the full 4.5 μs simulations. The red line marks the 5 Å threshold defining the bound regime. This plot provides additional insight into distance fluctuations and the spatial exploration of chemerin relative to CCRL2, illustrating the overall behavior of the system when the two proteins are not in contact. (D) Two-step model: The initial binding process of CCRL2 and chemerin in the 11 independent coarse-grained simulations. The blue chart represents the number of residue–residue contacts between chemerin and CCRL2_CRS1; the magenta chart shows the contacts between chemerin and CCRL2_CRS2. Contact occurs if the residue–residue distance is shorter than 5 Å. Simulation 6 is presented separately because its contact counts exceed 30, requiring a different *y*-axis scale from the other simulations (≤15). This plot illustrates that CCRL2–chemerin binding follows the two-step model, where the initial interaction occurs at the first chemokine-recognition site (CRS1), located in the N-terminal domain of the receptor, and a secondary contact occurs at the second chemokine-recognition site (CRS2), located in the extracellular loops.

### Energy Landscape Calculations

The free energy landscape (FEL), or potential of mean force, was computed as a function of the center-of-mass distance (d) and coordination number (coordinated) to characterize the distribution of conformational states. The rationale for this choice was to identify low-energy conformations where the proteins not only are spatially close but also establish stable intermolecular contacts. Considering the distance alone can produce states where CCRL2 and chemerin are adjacent yet weakly interacting. Therefore, the coordination number provides a quantitative measure of the contact surface, offering a more accurate depiction of the binding landscape. The Euclidean distance between chemerin and CCRL2 was computed by considering the center of mass (COM) of Cα atoms. The number of contacts between the backbone of chemerin (Group 1) and CCRL2 (Group 2) was calculated using a coordination function defined based on the number of atomic contacts between the two groups. The coordination number *c* was computed as

1 $$c=\sum_{i\in A}\sum_{i\in B}S_{ij}$$

where *s*_*ij*_ is a switching function that determines whether a contact between atoms *i* and *j* is formed. The function is given by

2 $$s_{ij}=1-{\left(\frac{r_{ij}-d_{0}}{r_{0}}\right)}^{n}/\left(1-{\left(\frac{r_{ij}-d_{0}}{r_{0}}\right)}^{m}\right)$$

where *r*_ij_ is the distance between atoms *i* and *j*, *d*_*0*_ is the reference distance set to 0.0 nm, *r*_0_ is the switching distance set to 0.9 nm, and *n* = 6 and *m* = 12 define the sharpness of the switching function. This approach allows for a continuous and differentiable coordination function that accurately captures interactions between chemerin and CCRL2, providing a reliable measure of their binding contacts during the simulation. All these operations were performed using the *driver* utility of PLUMED 2.8.^36^ An in-house Python script based on NumPy^37^ was used to compute the FEL as follows:

3 $$F\left(d,coord\right)=-k_{B}T\ln P\left(d,coord\right)$$

where *P* (*d*, *coord*) is the probability distribution function, *T* is the absolute temperature, and *k*_B_ is the Boltzmann constant. From this calculation, we obtained three representative structures: Conf1 (orange dot in Figure 2A and Supporting Information Figure S5A), Conf2 (blue dot in Figure 2A and Supporting Information Figure S5A), and Conf3 (green dot in Figure 2A and Supporting Information Figure S5A).

**Figure 2:**
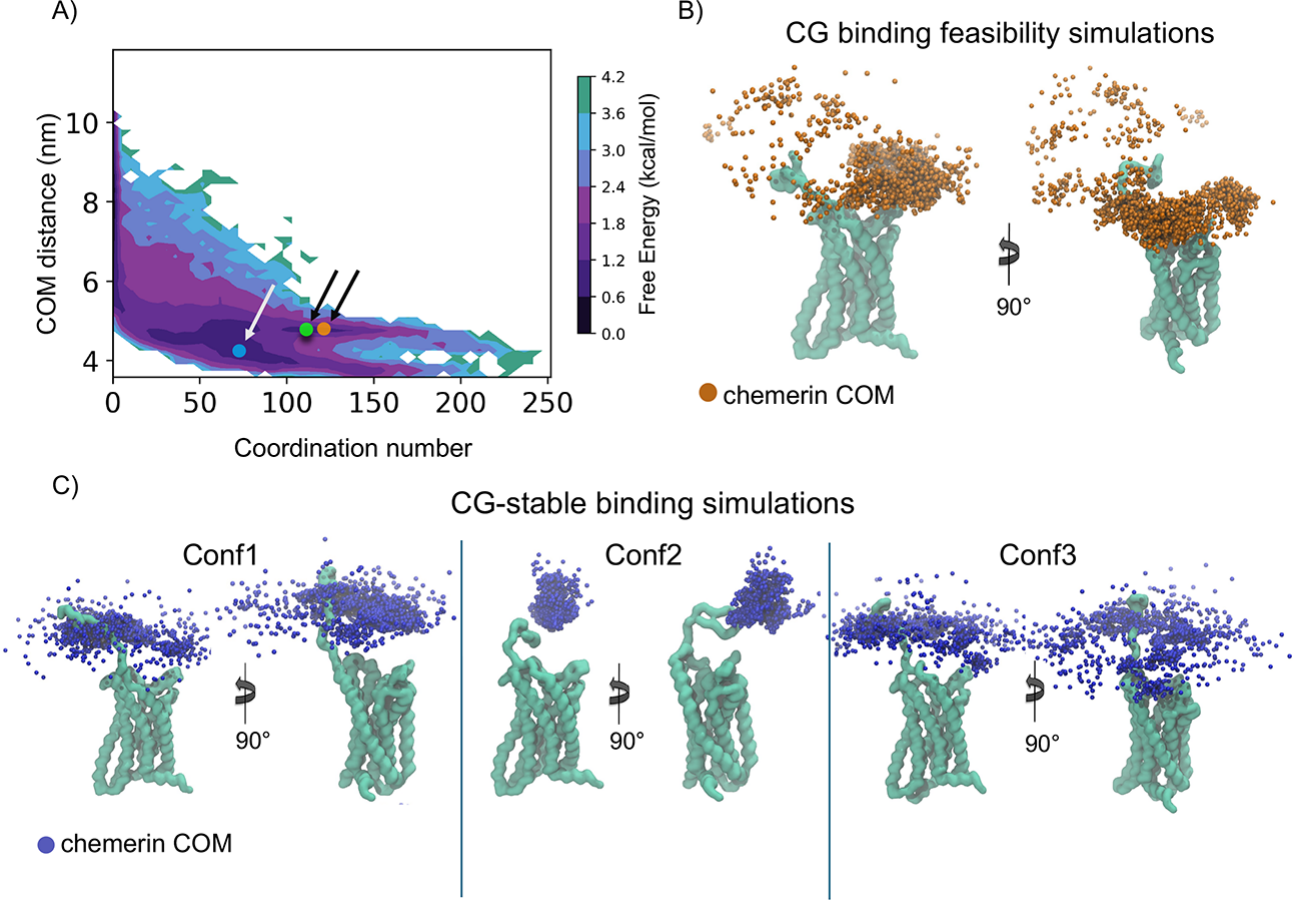
FEL and conformational dynamics of the CCRL2–chemerin complex. (A) Free energy surface (FEL) of the CCRL2–chemerin complex association across 11 simulations, plotted as a function of coordination and the COM distance between the two proteins. The lowest energy values are depicted in dark purple, transitioning through shades of purple, cyan, and green as the energy increases. Conformations corresponding to the lowest-energy regions of the FEL exhibit low distance and high coordination values. The structures corresponding to the three representatives of the most populated clusters (orange, blue, and green dots, respectively) within the main minima on the graph are marked by arrows. We will refer to these structures as Conf1, Conf2, and Conf3. (B,C) Structural representation of CCRL2, shown as a light green surface, with the COM positions of the chemerin core (excluding the C-terminal domain) represented as dots periodically extracted from MD trajectories. (B) Orange dots: COMs from all 11 replicas of the *CG-binding feasibility simulations*. (C) Blue dots: COMs from the 5 replicas of Conf1, 5 replicas of Conf2, and 5 replicas of Conf3 of the *CG-stable binding simulations*.

Because the combination of COM distance and coordination is orientation-agnostic, this description could, in principle, blur pose-specific minima. To evaluate whether this limitation influenced the interpretation of our results, we complemented the analysis with orientation-sensitive collective variables (CVs). Specifically, the global COM distance was replaced with the distance between the N-terminal domains of CCRL2 and chemerin, computed using the backbone atoms of residues 17–37 in CCRL2 and the first 10 backbone atoms of chemerin. This definition was motivated by previous findings indicating that the extracellular portion of the CCRL2 N-terminal domain contributes primarily to the initial recruitment phase rather than merely stabilizing the complex after binding is established. The distance between the N-terminal domains of CCRL2 and chemerin was calculated using an in-house Python script based on the MDTraj library.^38^

### CG-MD of CCRL2–Chemerin Complexes: Stable Binding Simulations

To investigate the stability of CCRL2–chemerin binding, we performed CG-MD simulations initiated from a bound configuration rather than from an unbound state where chemerin was positioned 30 Å from CCRL2. FEL calculations were carried out using all frames from 11 preliminary simulations, based on coordination number and interprotein distance metrics as described in the “Energy Landscape Calculations” Methods section.

We then performed cluster analysis on the conformations corresponding to the main FEL minima using the GROMOS algorithm implemented in GROMACS,^39^ applying a 0.9 nm RMSD cutoff. The three most populated clusters were identified, and their representative structures were selected. Clusters characterized by low interatomic distance and high coordination, indicative of stable, well-formed complexes, were prioritized, while those representing dissociated or low-contact states were excluded. The representative conformations from each of the three clusters were used to construct separate simulation systems. Using the INSANE script,^30^ coarse-grained models of CCRL2 and chemerin were embedded in a rectangular simulation box (15 × 15 × 20 nm^3^). The MARTINI3 force field parameters^31^ were employed, together with elastic network restraints applied to the backbone beads (force constant of 700 kJ/mol/nm^2^, cutoff distances 0.5–0.9 nm) to preserve the protein secondary and tertiary structure.^32^ The Conf1 system contained a total of 38319 atoms: 8065 POPC, 28319 water molecules, 406 Na^+^, 412 Cl^–^, and 1118 protein atoms. The Conf2 system contained 38264 atoms: 8077 POPC, 28252 water molecules, 406 Na^+^, 412 Cl^–^, and 1118 protein atoms. The Conf3 system contained 38268 atoms: 8077 POPC, 28256 water molecules, 406 Na^+^, 412 Cl^–^, and 1118 protein atoms.

System minimization and equilibration protocols were identical to those employed in the *binding_feasibility_simulations*, except for positional restraints on the N-terminal domain of CCRL2 during the production run. Each of the three representative conformations served as the starting point for five independent CG-MD replicas (5 × 3 μs), resulting in a total of 15 simulations and an aggregate simulation time of 45 μs. These simulations, referred to as *CG-stable_binding_simulations*, were initiated from different conformers and randomized initial velocities to ensure robust sampling of the bound state.

### MD Analyses

Trajectory analysis, including the calculation of minimum interatomic distances, root-mean-square deviation (RMSD) and root-mean-square fluctuation (RMSF), were performed using the built-in utility tools of GROMACS. RMSD calculations were performed to evaluate the fluctuations within the following regions: intraresidue chemerin, the C-terminal domain of chemerin, loop residues 72–97 of chemerin, intraresidue CCRL2, extracellular loop 2 (ECL2) of CCRL2, and the N-terminal domain of CCRL2.

All RMSD analyses for CCRL2 and chemerin were performed after aligning the trajectories to the innermost Cα atoms of their respective secondary structure elements (helices and β-strands), considering only frames corresponding to the CCRL2–chemerin bound state. These analyses were carried out across the three sets of MD simulations: *CG-binding_feasibility_simulations*, *CG-stable_binding_simulations*, and *AA-stable_binding_simulations*. For the *AA-stable_binding_simulations*, the RMSD of each individual transmembrane helix was also computed by fitting each frame to the internal portion of the corresponding helix and computing the backbone RMSD. This approach allowed us to identify the TM regions contributing to the internal mobility of CCRL2.

To monitor the spatial distribution of chemerin relative to CCRL2, the COM of the chemerin core (residues 1–280, excluding the flexible C-terminal domain) was calculated from 150 frames uniformly extracted along each MD trajectory and visualized as a dot. Results were visualized using VMD software.^40^

The RMSF, which quantifies the deviation of atomic positions from their average structure throughout the trajectory, was calculated using backbone atoms to quantitatively assess the magnitude of per-residue fluctuation in chemerin and CCRL2. RMSF analysis was performed on the three replicas of *AA-stable_binding_simulations* using the rmsf utility tool in GROMACS. The resulting RMSF values for each residue were then mapped onto the structures of the two proteins using a color gradient implemented through a custom Python script and ChimeraX-1.3 version.^41^

Electrostatic surface representations of CCRL2 and chemerin were generated using APBS within Chimera.^42^ Two clustering approaches were employed to analyze the MD trajectories: the GROMOS clustering algorithm^43^ implemented in GROMACS and the CloNe clustering tool.^44^ GROMOS-based clustering was performed separately on the combined 15 μs CG *stable binding simulations* for Conf1, Conf2, and Conf3, using system-specific RMSD cutoff of 1.5 nm for Conf1 and 1.2 nm for both Conf2 and Conf3, to ensure optimal clustering conditions.

CloNe clustering was applied to the all-atom (AA) stable binding simulations using features based on coordination and the COM distances between selected residues of chemerin and CCRL2. During the AA simulations, conformational rearrangements were observed that brought the N-terminal domain of chemerin into contact with the terminal residues of CCRL2’s N-terminal domain. This “flipping” event also facilitated the approach of the β1 strand of chemerin toward the ECL2 of CCRL2. To capture these dynamic events, the following residue ranges were selected for clustering: chemerin residues 21–31 and 50–65, and CCRL2 residues 24–35, 170–193, and 167–272. The COM distances and coordination values between these regions were calculated as described in the “Energy Landscape Calculations” section and used as input features for CloNe clustering.

### Analysis of Early Contact Dynamics between CCRL2 and Chemerin

Early contact dynamics between CCRL2 and chemerin were quantified using a two-stage analysis. First, a global contact analysis defined a binding event as any interaction distance <5 Å between any atom of CCRL2 and chemerin. Minimum interatomic distances were computed using the gmx mindist tool. This analysis was performed across 11 CG binding feasibility simulations as well as 15 simulations from the CG-stable binding set (5 replicas × 3 conformations).

Second, to gain further insight into the initial interaction dynamics, we conducted a region-specific analysis of the 11 selected replicas. Compared with the previous contact analysis, where we explored the potential binding between all regions of chemerin and CCRL2, this analysis focused on identifying which specific regions of CCRL2 interact first with chemerin. As in the previous approach, a distance of less than 5 Å was used as the interaction threshold. Minimum distances were calculated between all chemerin atoms and two functionally relevant regions of CCRL2: (i) the N-terminal domain (residues 1–31), and (ii) the extracellular loops (ECLs), comprising ECL1 (residues 93–103), ECL2 (residues 166–196), and ECL3 (residues 261–271). To probe temporal ordering between canonical recognition sites, we computed *z*-scored time-lag cross-correlation between CRS1 and CRS2 contact traces using custom Python scripts (NumPy^37^ and scipy.stats.zscore^45^). This procedure deemphasizes fleeting contacts, highlights persistent engagement patterns, and determines whether CRS1 tends to precede or follow CRS2 across replicas.

### CCRL2–Chemerin Contact Map and Statistical Distribution of Interactions

Contact maps for *CG-stable_binding_simulations* and *AA-stable_binding_simulation* were generated using in-house MDAnalysis script.^46^ The choice of distance cutoff followed established practices reported in the literature. For AAMD simulations, a cutoff distance of 4.0 Å was applied, as this value is commonly used to identify residue–residue contacts in atomistic representations.^38,47^ For CG-MD simulations, a slightly higher cutoff of 5.0 Å was employed to account for the lower spatial resolution and smoother potential functions of coarse-grained models, consistent with previous studies.^48^ For each frame, pairwise atomic distances between residues of CCRL2 and the chemerin were calculated using cdist function from SciPy.^45^ A contact was registered when any inter-residue distance fell below the selected cutoff. The resulting contact matrix was normalized by the total number of analyzed frames to obtain the percentage of frames in which each residue pair was in contact. The final percentage contact maps were visualized as heatmap, where higher percentage values indicate more persistent or recurrent. This analysis enabled the identification of hotspot residues critical for chemerin binding to CCRL2 and provided structural insight into the spatial organization of the ligand relative to the receptor.

Δ-Contact maps between Conf1, Conf2, and Conf3 were computed by first thresholding each contact matrix at 10%, retaining residue pairs that were in contact for >10% of the simulation frames. The thresholded contact matrices for Conf1 and Conf3 were then compared pairwise (Conf1–Conf2, Conf2–Conf3, Conf1–Conf3) by subtraction. Positive values in the Δ-map indicate contacts enriched in the first conformation, while negative values correspond to contacts enriched in the second conformation.

The statistical distribution of interaction frequencies (expressed as the percentage of the total simulation time) between CCRL2 and chemerin was quantified using ProLIF^49^ on 1/3 of the total simulation frames. The ProLIF Fingerprint module was employed to detect and characterize key interaction types including hydrophobic contacts, hydrogen bond donors and acceptors, π-stacking, cation–π interactions, ionic (anionic and cationic) interactions, and vdW contacts. Interaction frequencies were computed as the percentage of frames in which each interaction was detected, allowing a quantitative assessment of the most persistent molecular contacts. To further elucidate the CCRL2–chemerin binding interface, interactions between specific structural regions identified from the contact map were analyzed. These included: (i) the N-terminal domains of CCRL2 and chemerin; (ii) the ECL2-β1 interface involving chemerin residues 58–61 and CCRL2 residues 171–174, 185, and 188–191; (iii) the N-terminal-loop3 interface, involving chemerin residues 71, 82, 90, 91, and 93–95 with CCRL2 residues 6, 8, 9, and 11; and (iv) the ECL2-TM5-C-terminal interface, which involves chemerin residues 148–157 and CCRL2 residues 168–171 and 189–199.

Interactions were classified into functional categories, hydrogen bonds (HBDonor and HBAcceptor), π-interactions (π-stacking and cation–π), electrostatic interactions (anionic and cationic), and vdW contacts, to facilitate a detailed quantitative and structural analysis of the binding mechanism. The results were visualized using color-coded bar plots to highlight region-specific interaction patterns and their relative persistence throughout the simulation.

Finally, representative interactions between chemerin and CCRL2 were analyzed in the most populated cluster using the protein–ligand interaction profiler.^50^

### CCRL2–Chemerin All-Atom Stable Binding MD Simulations

The conformation corresponding to the representative structure of the most populated cluster from the Conf2 binding stability simulations was selected as the starting model for all-atom MD. This choice was based on comparison among the cluster representatives from Conf1, Conf2, and Conf3 simulations, which showed that Conf2 and Conf3 adopted identical orientation. In two of the three cases, these orientations correspond to the most frequently sampled configuration during the simulations. To further validate this selection, principal component analysis (PCA) was performed on global structural descriptors extracted from the full AA trajectories (see the *Principal Component Analysis of Binding Conformations* section). The selected conformation was backmapped to an AA representation using the CHARMM-GUI Martini Maker’s all-atom converter.^51,52^ The CCRL2–chemerin system was then prepared using the CHARMM-GUI membrane builder platform.^46,53–59^ AAMD simulations in explicit solvent were performed with the GROMACS version 2022.3.^33^ The CHARMM36m force field^60^ was used to describe interatomic forces, and TIP3P water was employed for solvation^61^ in a rectangular box with PBCs, maintaining a 10 Å boundary. A buffer of 0.15 M NaCl was added to ensure the system is electrically neutral. The resulting system contained 414,036 atoms, including 41,674 POPC atoms, 109 Na^+^ ions, 117 Cl^–^ ions, 120,378 water atoms, and 7461 protein atoms (chemerin and CCRL2).

The N-terminal residue of chemerin was capped with an acetyl group, and the C-terminal residues of both chemerin and CCRL2 was methylated. Protonation states of histidine residues were determined at pH 7 using PropKa,^62^ which predicts the p*K*_a_ values of ionizable groups based on their local environment. Histidine residues were assigned as δ-protonated: H38, H40, H116, H130, and H146 in chemerin and H96, H134, H198, H266, H283, H292, and H316 in CCRL2. Chemerin was modeled with disulfide bonds C98–C117 and C101–C135, consistent with the two most recent cryo-EM full-length structure,^18,27^ and C77–C87, as reported in UniProt but unsolved in one cryo-EM structure^18^ due to missing residues 86–95 present in the alternative cryo-EM structure.^27^ CCRL2 was modeled with a disulfide bond between C103 and C181.

EM was performed in two stages using the steepest descent algorithm: an initial minimization of 10 steps for 19 iterations followed by an additional minimization of 5000 steps to remove steric clashes. Harmonic position restraints were applied as follows: 3000 kJ/mol·nm^2^ on the CCRL2 and chemerin backbone atoms, 3500 kJ/mol·nm^2^ on the side chains of chemerin and CCRL2, 3500 kJ/mol·nm^2^ on P atoms of the lipid headgroups, and 3500 kJ/mol·nm^2^ and 3500 kJ/mol·nm^2^ on two dihedral angles, one formed by C1–C3–C2–O21 and C28–C29–C210–C211 atoms.

Following minimization, systems were initialized with randomly assigned velocities and equilibrated through seven cycles, each consisting of 3 ns of NPT equilibration followed by 3 ns of NVT equilibration except for the final cycle (7 ns NPT + 7 ns NVT), yielding a total equilibration time of 50 ns. The integration time step was 1 fs for the initial cycles and 2 fs for the last six. Temperature coupling at 300 K was achieved using the v-rescale thermostat,^35^ and pressure coupling at 1 atm was controlled using the stochastic C-rescale barostat.^63^ During equilibration, the system’s temperature was gradually increased from 80 to 300 K. Restraints were applied to the backbone and side-chain atoms of CCRL2 and chemerin, the phosphorus (P) atoms of the lipid headgroups, and the two POPC dihedral angles. These restraints were progressively reduced across the equilibration cycles.

Production runs, without positional restraints, were performed by using an integration time step of 2 fs. Initial velocities for the systems were randomly chosen from a Maxwell distribution at 300 K. Hydrogen bonds were constrained using linear constraints solver,^64^ and long-range electrostatic interactions were computed using the PME method^34^ with a cutoff of 1.2 nm. The same cutoff was applied to short-range electrostatic interactions and Lennard-Jones interactions. All equilibration and production simulations were carried out with GROMACS 2022.3.^33^ Three independent 1.5 μs production simulations were performed for a total of 4.5 μs. All input parameters required to reproduce the simulations are provided in the file input_MD.zip, available in the Supporting Information.

### Principal Component Analysis of Binding Conformations

To quantify the similarity between the three coarse-grained binding modes (Conf1 and 3), PCA was performed on global structural descriptors extracted from the full CG trajectories, including (i) the center-of-mass distance between chemerin and CCRL2, (ii) the total number of intermolecular contacts, and (iii) the orientation angle between chemerin and CCRL2, defined by vectors connecting N- to C-termini. For each ensemble, we computed (i) the Euclidean distance between PCA centroids and (ii) the Jensen–Shannon divergence (JSD) between their probability distributions in PCA space.^65,66^ The resulting low-dimensional embedding allowed a comparison of conformational ensembles. Conf2 and Conf3 occupied largely overlapping regions in the PCA space, whereas Conf1 was separated. In agreement with the Δ-contact map analysis, these results indicated that Conf2 and Conf3 correspond to highly similar binding poses that are distinct from Conf1. Consequently, the representative structure of the most populated Conf2 cluster was selected as the starting configuration for subsequent AA-MD simulations.

### Analysis of Missense Variants in the Context of CCRL2–Chemerin and Chemerin–CMKLR1 Complexes

Missense variant data were obtained from the gnomAD database (v4, GRCh38).^67^ Variants were prioritized using AlphaMissense,^68^ an algorithm that integrates structural context and evolutionary conservation to estimate the potential pathogenicity of amino acid substitutions. To evaluate the functional relevance of these variants, AlphaMissense scores were cross-referenced with contact maps derived from structural models of the CCRL2–chemerin (Supporting Information Figures S14 and S15) and chemerin–CMKLR1 (Supporting Information Figure S16) complexes. Interaction hotspots were identified from CCRL2–chemerin MD simulation (Figure 4) and the cryo-EM structure of the chemerin–CMKLR1 complex (PDB ID: 8ZJG).^27^ Candidate missense variants were further analyzed based on the biochemical properties of the substituted residues, focusing on those classified as “likely pathogenic” or “ambiguous.”

The effects of missense mutations on the CCRL2–chemerin and CMKLR1–chemerin complexes were quantified as a change in Gibbs free energy (ΔΔ*G*) between the wild-type and mutant complexes, computed using FoldX tool (version 5.1).^69^ The RepairPDB, BuildModel, and AnalyzeComplex commands were applied sequentially. The RepairPDB identifies residues with unfavorable torsion angles, vdW clashes, or high total energies and corrects them; this step is recommended before performing FoldX calculations. The BuildModel introduces specific point mutations and generates corresponding mutant structures, while AnalyzeComplex calculates the interaction energy between molecular partners. Additional ΔΔ*G* values for the CCRL2–chemerin complex were obtained using Mutabind2,^70^ DynaMut2,^71^ and DDMut-PI^72^ tools. In FoldX and MutaBind2, positive ΔΔ*G* values indicate destabilizing mutations, whereas in DynaMut2 and DDMut-PPI, negative ΔΔ*G* values denote destabilization. Energy thresholds for assessing the protein stability were set to >2 kcal/mol for destabilizing and <−2 kcal/mol for stabilizing mutations, consistent with previously reported criteria.^73–75^

To further validate the FoldX results at the structural level, residue-wise interaction and network parameters were analyzed using the Protein Contacts Atlas web tool.^76^ For each residue, values of solvated area, degree, betweenness centrality, and closeness centrality were retrieved, representing, respectively, the degree of solvent exposure, the number of direct residue–residue contact, the residue’s role in connecting network nodes, and its centrality in the network itself. Finally, known pathogenic missense variants were identified by querying multiple variant databases and verified trough ClinVar,^77^ a curated repository that aggregates information on the clinical significance of genomic alterations.

### Template-Based Reconstruction of the CCRL2–Chemerin–CMKLR1 Ternary Complex

The CCRL2–chemerin–CMKLR1 ternary complex was reconstructed using a template-based modeling strategy, integrating experimental and simulation data. The cryo-EM structure of the CMKLR1–chemerin complex (PDB: 8ZJG) was combined with the representative structure from the most populated cluster of the first replica of our AA simulation of stable binding. This CCRL2–chemerin conformation was selected because chemerin adopted an orientation conducive to a simultaneous engagement with CMKLR1. To align the two components, we superimposed the Cα atoms of chemerin from the cryo-EM structure onto those of chemerin from the MD-derived CCRL2–chemerin complex. Because the cryo-EM structure lacks several flexible loops that our simulations indicate are important for CCRL2 binding, these regions were rebuilt using Modeller 10.4.^78^ We also refined the position of the N-terminal domain based on the simulation data to obtain a more physiologically relevant orientation. Dual-template modeling in Modeller used: (i) the cryo-EM structure of chemerin (PDB ID: 8ZJG), excluding the first 10 N-terminal residues, and (ii) the simulation-derived chemerin structure, omitting the last 20 C-terminal residues. The resulting hybrid model, hereafter defined *Chemerin_remodelled*, replaced the chemerin coordinates in both component structures and yielded a plausible of the full CCRL2–chemerin–CMKLR1 ternary complex (Supporting Information Figure S19), illustrating how chemerin may bridge the two receptors.

## Results and Discussion

### Chemerin Spontaneously Binds CCRL2

To investigate the binding mechanism between chemerin and CCRL2, we performed 26 independent coarse-grained molecular dynamics (CG-MD) simulations totaling approximately 80 μs of simulation time. The CCRL2–chemerin complex was initialized with CCRL2 embedded in a membrane with an extended CCRL2 N-terminal region, while chemerin’s N-terminal portion was positioned 30 Å away from CCRL2 (measured from the first methionine of CCRL2) (Supporting Information Figure S2). In 9 out of 26 simulations, no interaction was observed. In 6 simulations, chemerin transiently interacted with CCRL2, exhibiting repeated association and dissociation events but without forming stable contacts sufficient to identify key stabilizing residues. The remaining 11 simulations showed sustained chemerin–CCRL2 interactions and are hereafter referred to as *CG-binding_feasibility_simulations*. We analyzed the minimum interprotein distance across the 11 selected simulations to distinguish bound from unbound states (Figure 1A–C).

Approximately 50% of all frames exhibited close contacts, indicating a stable binding interface. Notably, 4 out of the 11 selected simulations (i.e., *CG-binding_feasibility_simulations* 5–8) showed extended interaction periods, whereas the remaining cases displayed reduced persistence and stability in trajectories terminated at 3 μs, and no or only sporadic contacts in those stopped at 1.5 μs (Figure S2B–E). Further details and the rationale behind this selection are provided in the Methods section. Together, these results demonstrate that chemerin can spontaneously bind to CCRL2 and highlight the flexible and dynamic nature of the interaction.

### The N-Terminal Region of CCRL2 Drives Initial Chemerin Binding Dynamics

Following our observation that chemerin can spontaneously associate with CCRL2 (Figure 1A–C), we next aimed to identify which regions of CCRL2 are primarily responsible for the initial interaction with chemerin. According to the well-established two-site/two-step model of chemokine receptor activation,^79,80^ the initial binding involves interaction between the chemokine core and the receptor’s N-terminal region (Chemokine Recognition Site 1, CRS1), followed by engagement of the chemokine N-terminus with the receptor’s extracellular or transmembrane regions (Chemokine Recognition Site 2, CRS2), ultimately leading to receptor activation. In this framework, to investigate the distinct interaction patterns between chemerin and the N-terminal region or extracellular loops (ECLs) of CCRL2, we designed the N-terminal region of CCRL2 as *CCRL2_CRS1* and its three extracellular loops as *CCRL2_CRS2* (Figure 1B).

To dissect this two-step binding mechanism in the CCRL2–chemerin complex, we refined our analysis strategy. Instead of quantifying the minimum distance between any CCRL2 and chemerin atoms (Figure 1A), we measured contact frequency (interatomic distances ≤ 5 Å) between chemerin and the defined *CCRL2_CRS1* and *CCRL2_CRS2* regions (Figure 1D). In 10 out of 11 CG-MD simulations, chemerin consistently interacted with *CCRL2_CRS1* prior to engaging *CCRL2_CRS2* (Figure 1D), suggesting that the N-terminal region may play a key role in the initial recruitment of chemerin. To more rigorously examine the temporal order of these events, we applied z-scored time-lag cross-correlation analysis, which emphasizes persistent engagement and minimizes the influence of fleeting single-frame contacts visible in raw traces (Supporting Information Figure S3). Within this framework, a negative lag at the correlation maximum indicates that *CCRL2_CRS1* engagement precedes *CCRL2_CRS2*, whereas a positive lag suggests the reverse order. Using this approach, 7 out of 10 simulations exhibited a negative peak, supporting a *CCRL2_CRS1→CCRL2_CRS2* binding sequence; in the remaining simulations, both regions appeared to engage nearly simultaneously or with *CCRL2_CRS2* preceding *CCRL2_CRS1*. Taken together, these results suggest that CCRL2 likely follows a two-step binding mechanism analogous to those of other GPCRs, with CRS1 generally acting as the initial recruitment site.

To further characterize CCRL2 conformational flexibility during binding, we computed the RMSD distributions for the N-terminal region and extracellular loop 2 (ECL2) in both bound and unbound states. Unexpectedly, chemerin binding did not reduce RMSD values in either region (Supporting Information Figure S4), suggesting that chemerin remains in a transient or exploratory binding mode without adopting a fully stabilized conformation. Although our simulations demonstrate spontaneous chemerin association with CCRL2 via CRS1, they do not capture a stable, well-defined interface, likely reflecting a dynamic and potentially reversible binding process.

### CCRL2–Chemerin Complex: From Initial Contact to Stable Complex Formation

To overcome the limitations associated with the transient interactions and more accurately characterize key binding events and stabilizing contacts within the CCRL2–chemerin complex, we extended our coarse-grained molecular dynamics CG-MD simulations by selecting representative conformations of bound states (Supporting Information Figure S5A). A bidimensional free energy surface (2D-FEL) was computed to evaluate the stability of the complex’s conformational states. The FEL was constructed as a function of the distance between the centers of mass (COM) of CCRL2 and chemerin and their coordination number, as sampled across the *CG-binding_feasibility_simulations* (Figure 2A).

Energy minima in the FEL corresponded to stable bound states, characterized by high coordination number (i.e., many atomic contacts between CCRL2 and chemerin) and short interprotein distances (reflecting proximity between the two proteins). Clustering of these minima identified three highly populated states (clusters) and representative protein structures (Conf1–3).

To evaluate whether the use of orientation-agnostic collective variables (CVs; COM distance and coordination) introduced degeneracy, we repeated the analysis using an orientation-sensitive CV, the N-terminal–to–N-terminal distance, in combination with coordination (Supporting Information Figure S5B). The three lowest-energy representatives obtained with this alternative FEL were compared with those derived from the original FEL (Supporting Information Figure S5C). For clarity, we refer to these representative conformations as Rappr1, Rappr2, and Rappr3 across both analyses.

Overall, the conformations extracted from the two approaches were broadly consistent. Apart from Rappr3, which displays a slightly greater structural divergence, the representative conformations were highly similar. Importantly, in both sets of minima, a conserved trend in the orientation of the chemerin’s N-terminus (highlighted in red) was observed: in Rappr1, it is oriented outward, whereas in Rappr2 and Rappr3, it was directed toward the CCRL2 N-terminal. This agreement indicates that while the inclusion of orientation-sensitive CVs enhances structural discrimination, the essential binding orientation is preserved across both FEL definitions. Consequently, the conformations derived from COM distance and coordination were considered equivalent, for the purpose of extended CG simulations, to those identified using orientation-sensitive CVs.

Together, these results validate Conf1, Conf2, and Conf3 as reliable representatives of bound states, which were subsequently used as starting points for 15 additional CG-MD (3 × 5 μs replicas per cluster), termed *CG-stable_binding_simulations.*

To validate whether these new simulations captured more stable CCRL2–chemerin binding, we compared internal fluctuations across data sets. RMSD analysis of CCRL2’s TM helices revealed well-equilibrated states in both simulation sets (Supporting Information Figure S6A). Notably, lower RMSD values were observed for chemerin’s internal fluctuations in the *CG-stable_binding_simulations* compared to the initial *CG-binding_feasibility_simulations* (Supporting Information Figure S6C-I and D-I), particularly in loop2 and the C-terminal domain (Supporting Information Figure S6C-II, S6C-III and S6D-II and S6D-III). Additionally, reduced fluctuations were seen in CCRL2’s ECL2 (Supporting Information Figure S6B) and CCRL2’s N-terminal (Supporting Information Figures S6C-IV and S6D-IV), suggesting a stabilized binding interface.

To further assess the positional stability of chemerin relative to CCRL2, we tracked the center of mass (COM) of the chemerin’s core extracted periodically from MD trajectories, considering only on bound frames. In the *CG-binding_feasibility_simulations*, chemerin explored a broad region around CCRL2, including initial contacts with its N-terminal domain (Figure 2B, orange dots). In contrast, in the *CG-binding_stability_simulations*, chemerin remained more localized around CCRL2, exhibiting fewer binding–unbinding events (Figure 2C, blue dots). In particular, in the Conf2-derived simulations, chemerin remained firmly attached, as indicated by a dense cluster of COM points localized on the helical bundle of CCRL2.

To validate these findings, FEL landscapes were recalculated from the *CG-stable_binding_simulations* using COM distance and coordination number as reaction coordinates. For each of the 3 starting clusters, energetic minima from the 5 simulation replicas were projected onto the FEL (black arrows, Figure 3A).

**Figure 3:**
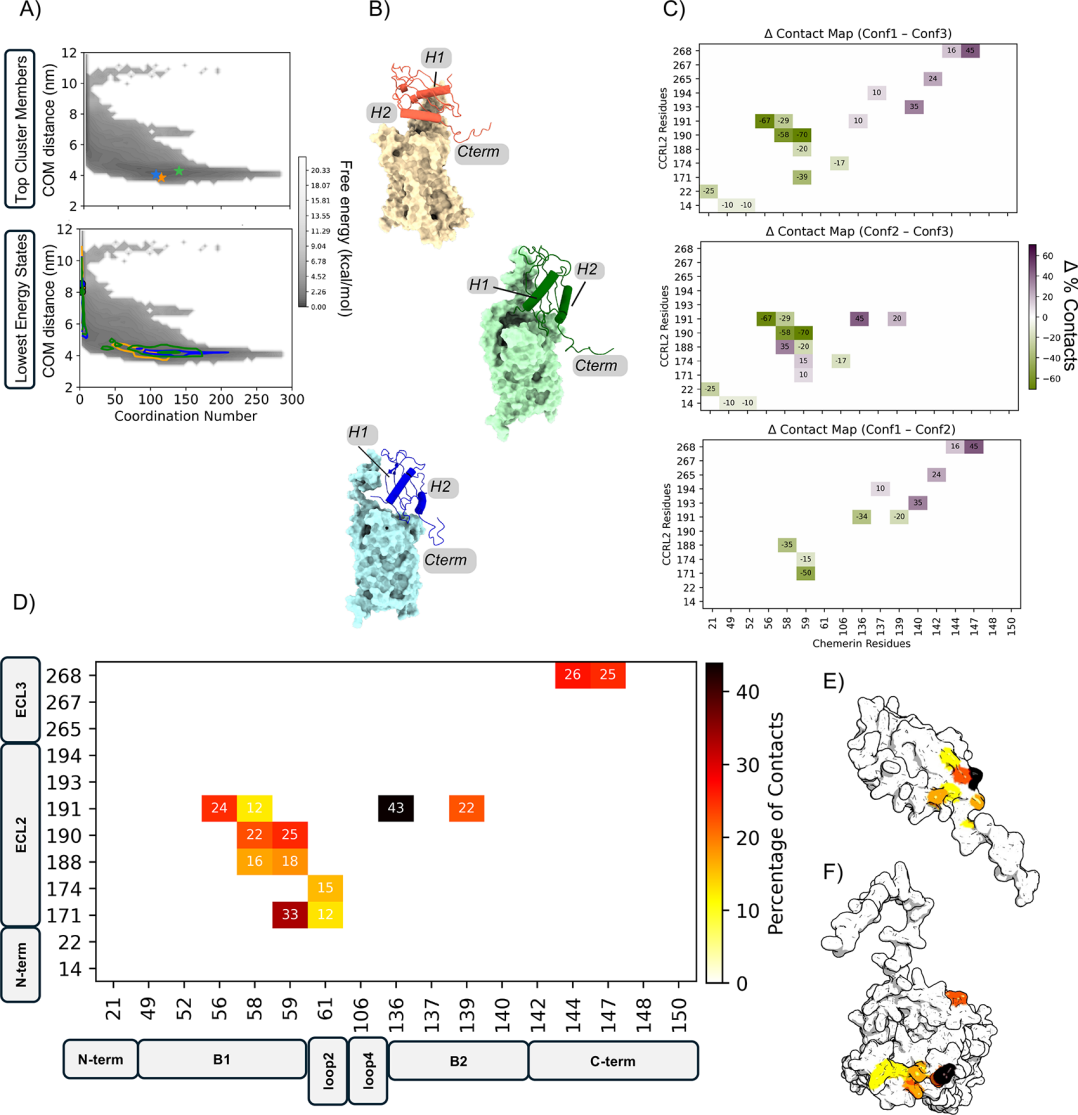
Comparative of Conf1–Conf2–Conf3 simulations. (A—top panel) FEL plots of Conf1–Conf3 across 5 replicas, with lowest-energy conformations indicated by color-coded dots (orange, blue, green). The FEL is depicted in grayscale, from dark to light, representing increasing energy levels. Darker regions correspond to low-energy conformations characterized by low distance and high coordination, indicating that the two proteins are bound to each other. (A—bottom panel) FEL plots of Conf1–Conf3 across 5 replicas, with cluster projections of the most populated conformations. Multiple colored dots represent the coordination and distance values of the most populated cluster members across the concatenated replicas for each conformation (orange for Conf1, blue for Conf2, and green for Conf3). (B) All-atom models of representative structures for each Conf, showing CCRL2 (light surface) and chemerin (dark cartoon). (C) Differential contact maps between the simulated conformations (Conf1–Conf3, Conf2–Conf3, and Conf1–Conf2). Contacts unique to the first conformation in each pair are shown in purple, while contacts unique to the second conformation are shown in green. The top panel shows Conf1–Conf3, the central panel Conf2–Conf3, and the bottom panel Conf1–Conf2. (D) Weighted contact maps of CCRL2–chemerin interfaces, retaining contacts present in >10% of frames. (E,F) Surface representations of chemerin and CCRL2, respectively, colored by contact frequency.

The minima identified in each replica consistently overlapped with the most populated clusters (Figure 3A), supporting the robustness of our ensemble-based strategy. This ensemble-based approach enabled refined characterization of the CCRL2–chemerin binding interface without relying on a single structural model.

Contact frequency maps (Figure 3D and Supporting Information Figure S7) were generated from representative conformations across all 15 simulations, providing a statistically robust basis for identifying key interactions. Only persistent contacts (present in more than 10% of frames) were considered, thereby excluding transient interactions.

The β1 strand and adjacent loop2 of chemerin, particularly residues V56, T58, P59, and P61, emerged as primary contact points, engaging CCRL2’s ECL2 residues K171, M174, F188, P190, and A191. Additional interactions involved chemerin’s helix H2 residues L136 and Q139 with CCRL2 ECL2 and residues D144 and S147 with CCRL2 ECL3 residues F267 and S268 (Figure 3E,F).

These findings are consistent with experimental evidence, indicating that chemerin predominantly engages CCRL2 via its N-terminal region. This is consistent with prior observations indicating that the C-terminal nonapeptide of bioactive chemerin does not compete with full-length chemerin for CCRL2 binding.^5^ In addition, the absence of stable interactions involving chemerin’s C-terminal portion supports the specificity of this binding mode.^2^

### All-Atom MD Simulations Reveal Stable CCRL2–Chemerin Interactions Mediated by ECL2−β1 Contact and N-Terminal Anchoring

To gain atomic-level insights into the molecular interactions within the CCRL2–chemerin complex, we performed all-atom molecular dynamics (AA-MD) simulations (hereafter referred to as *AA-stable_binding_simulations*), initiated from the most populated cluster identified in the *CG-stable_binding_simulations* (see Methods for details). Among the three coarse-grained binding modes (Conf1–3), the representative conformation of Conf2 was selected as starting structure. This choice was motivated by a comparative analysis of the conformational ensembles: Δ-contact maps highlighted that Conf2 and Conf3 shared highly similar interfacial contacts, in contrast to Conf1, which displayed a distinct binding fingerprint (Figure 3C). Consistently, PCA in a global descriptor space (center-of-mass distance, intermolecular contacts, orientation angle) showed that Conf2 and Conf3 occupied overlapping regions of conformational space, while Conf1 was clearly separated (Supporting Information Figure S7D). Quantitatively, the pairwise Euclidean distances between the centroids of the conformational ensembles in PCA space supported this conclusion: Conf2 and Conf3 were much closer to each other (distance = 0.073) than either was to Conf1 (Conf1–Conf2 = 0.452; Conf1–Conf3 = 0.525). Similarly, the JSD between the Conf2 and Conf3 distributions (0.37) was substantially lower than that involving Conf1, when compared with both Conf2 (0.73) and Conf3 (0.70), further indicating that Conf2 and Conf3 converge toward a common binding orientation distinct from Conf1.

Using Conf2 as the back-mapped all-atom starting structure, we carried out 1.5 μs-long AA-MD simulations across three independent replicas. The time evolution of backbone RMSD confirmed that all simulations were equilibrated (Supporting Information Figure S8A,B). As shown in Supporting Information Figure S8G, which reports the COM of the chemerin core, chemerin remained stably associated with CCRL2 in the *AA-stable_binding_simulations*, exhibiting markedly reduced positional deviations compared with the *CG-binding_feasibility_simulations* and the *CG-stable_binding_simulations* (Figure 2B,C).

The largest RMSD fluctuations were observed in CCRL2’s N-terminal domain and extracellular loop 2 (ECL2) (Supporting Information Figure S8C,D) and in chemerin’s β2−β3 loop and C-terminal region (Supporting Information Figure S8E,F). Residue-specific flexibility, assessed via root-mean-square fluctuations (RMSF), revealed high mobility in chemerin loops 1 (residues 40–58), 2 (72–96), and 3 (116–125) and its C-terminal region (141–157). In CCRL2, the most flexible regions were the N-terminal portion, the first 20 residues of TM1, and helices TM5 and TM6 (Supporting Information Figure S9A,B).

To further characterize the CCRL2–chemerin interaction interface, we mapped persistent contacts observed throughout the *AA-stable_binding_simulations* (Figure 4 and Supporting Information Figure S10).

**Figure 4:**
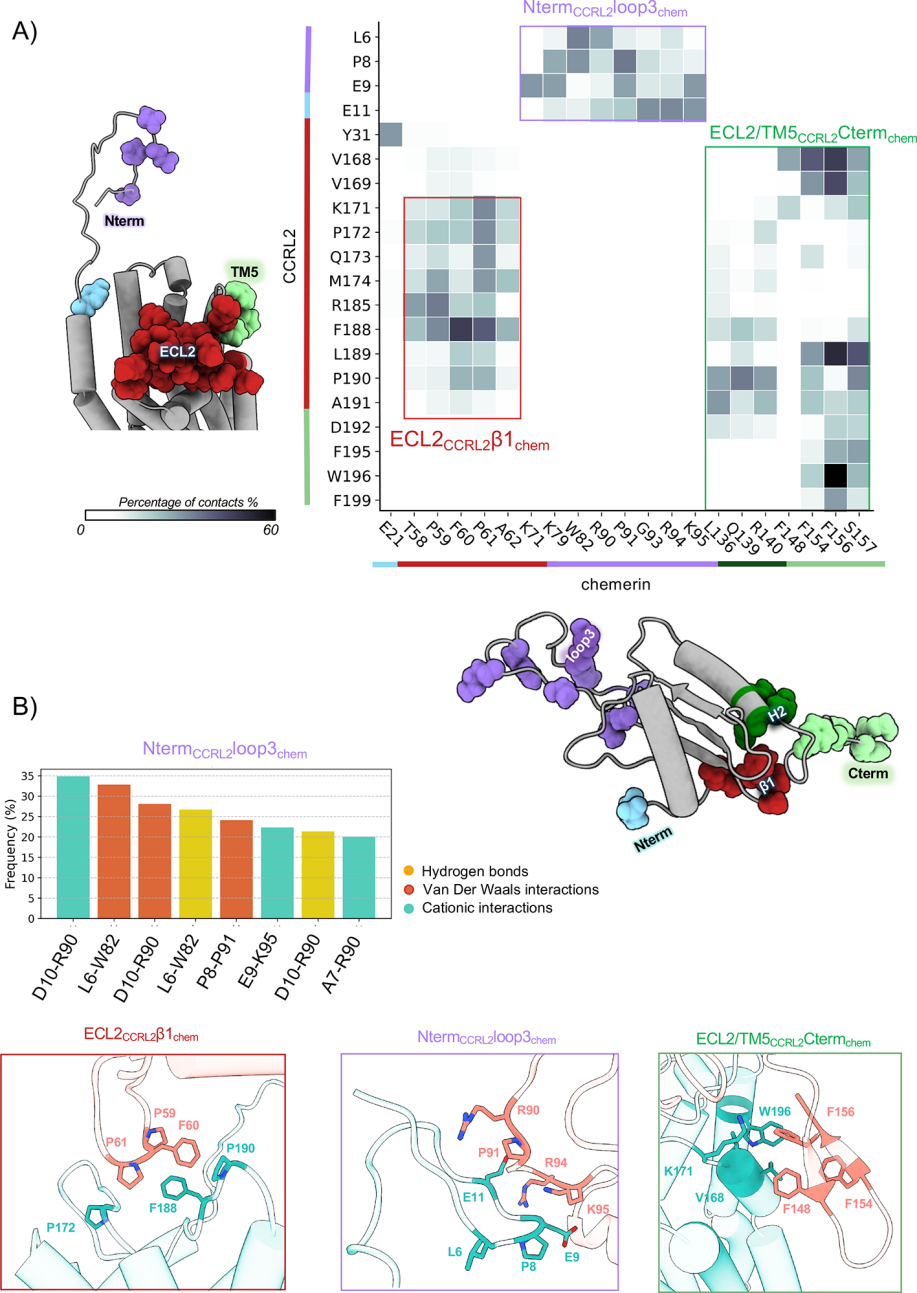
Structural and dynamic characterization of CCRL2–chemerin interactions from MD simulations. (A) Heatmap of contact frequencies between CCRL2 and chemerin residues over the full 1.5 μs trajectory (concatenated replicas), showing only contacts persisting >25% of the time. Contact regions are annotated with color-coded structural bars and mapped onto 3D models. (B) Representative interaction pairs from each contact region are shown as cartoon and stick models. Adjacent bar plots show the percentage of simulation time during which specific interactions occurred, categorized as vdW (red), hydrogen-bond donor (yellow), and cationic (light blue). Only interactions persisting for >20% of the trajectory are reported.

This analysis revealed three major interaction regions, consistent with those identified in the *CG-stable_binding_simulations.* The first region involves ECL2 of CCRL2 and the β1 strand of chemerin (ECL2_CCRL2_–β1_chem_), the second involves the N-terminal of CCRL2 and chemerin loop 3 (Nterm_CCRL2_–loop3_chem_), and the third is centered on the interactions between ECL2/TM5 of CCRL2 and the chemerin C-terminal (ECL2/TM5_CCRL2_–C-term_chem_) (Figure 4A,B). The following paragraphs describe each of these interaction regions in detail.

### CCRL2–Chemerin Binding via ECL2−β1 Contact Region Defines a Stable Interaction Interface and Modulates Chemerin N-Terminal Binding Modes

The primary interface (ECL2C_CRL2_–β1_chem_; CRS2 in the canonical two-site model) involved chemerin residues D57–A62 and CCRL2 residues K171, P172, Q173, M174, R185, F188, L189, P190, and A191. The most frequent contacts (see Methods for contact definition) were F188_CCRL2_–F60_chem_ (50% of the simulation time), F188_CCRL2_–P59_chem_ (31%), K171_CCRL2_–P61_chem_ (31%), and R185_CCRL2_–P59_chem_ (35%). Additional interactions, such as R185_CCRL2_–T58_chem_ (27%) and M174_CCRL2_–P61_chem_ (27%), also contributed to interface stability. Stabilization arose from vdW and hydrogen bond interactions (K171_CCRL2_–P61_chem_, R185_CCRL2_–P59_chem_, and M174_CCRL2_–P59_chem_), π–π stacking (F188_CCRL2_–F60_chem_), and a putative cation–π interaction (K171_CCRL2_–F60_chem_) (Figure 4A,B and Supporting Information Figure S10).

To further characterize this interface, we quantified the COM distance and coordination number for residues in ECL2_CCRL2_–β1_chem_ interaction (Supporting Information Figure S10 A) and performed two-dimensional clustering with the density-based CLoNe algorithm (Supporting Information Figure S11B–D).^44^ Contour plots revealed diverse binding behaviors across the three all-atom MD replicas. Because replicas may not provide statistically independent results, we analyzed them separately to capture binding modes and transient conformational states that could be obscured by aggregate analysis. Representative complexes from the most populated cluster were characterized by coordination = 78, COM distance = 12 Å (replica 1), coordination = 60, COM distance = 14 Å (replica 2), and coordination = 25, COM distance = 23 Å (replica 3), indicating progressively weaker interfacial engagement (Figure 5A–C).

**Figure 5:**
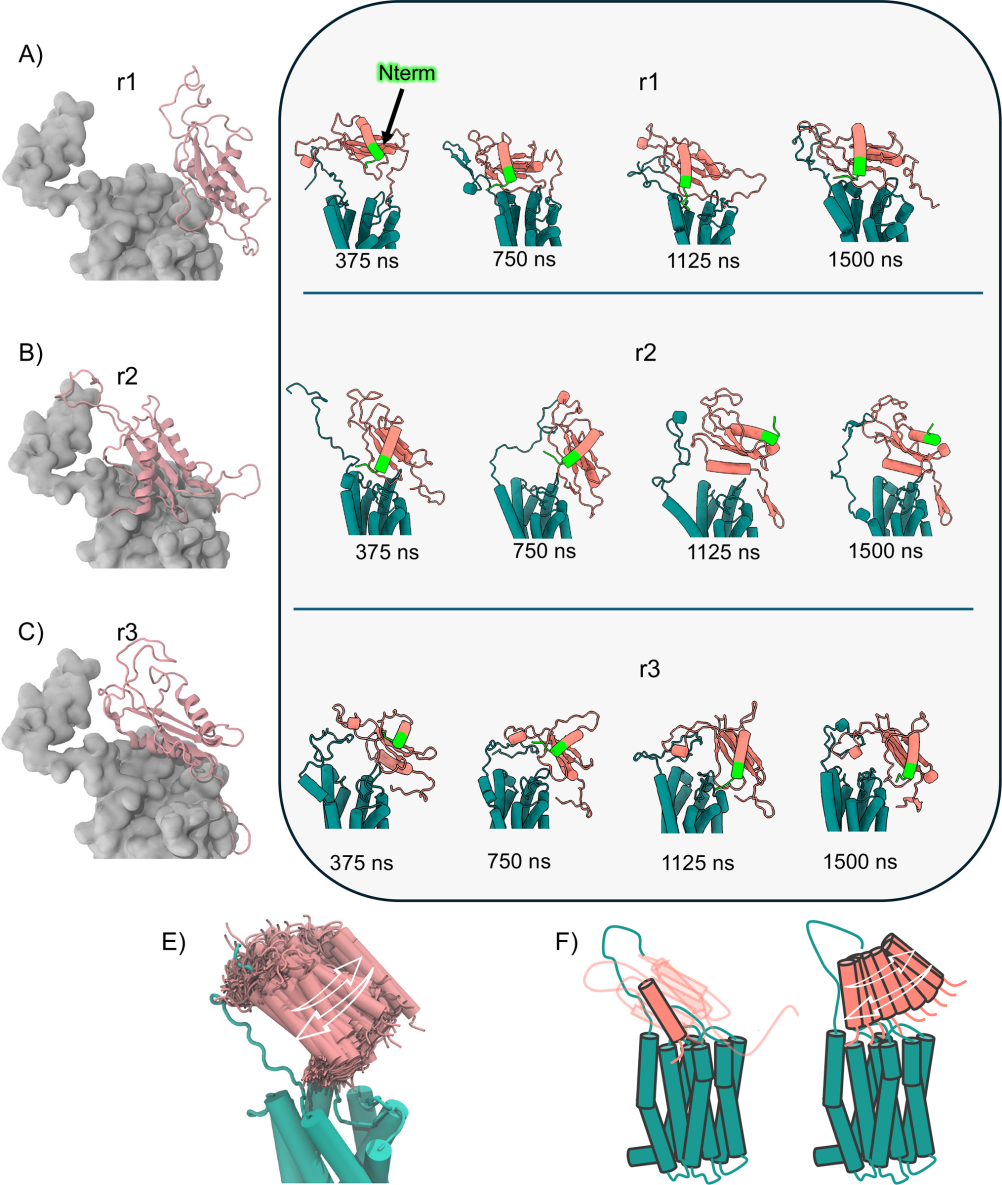
Insights into chemerin–CCRL2 interactions: structural clustering, binding evolution, and N-terminal conformational dynamics. (A–C) Structures of the most populated clusters from each replica. CCRL2 is shown as a gray surface, and chemerin is rendered in light pink cartoon. (D) Time-resolved snapshots (one per 175 ns) over 1.5 μs showing the evolution of chemerin binding. Chemerin’s N-terminal region is highlighted in lime. (E) Superimposed snapshots from the first 500 ns of replica 1 (500 frames) showing conformational changes of the chemerin N-terminal domain. This visualization highlights the progressive interaction dynamics between chemerin and CCRL2, particularly the N-terminal anchoring mechanism. (F) Schematic representation of the dynamic range of N-terminal conformations. Left: α1 helix and N-terminus orientation; right: approach of the N-terminal domain and α1 helix toward CCRL2’s TM1 and N-terminal region as observed in the MD simulations of replicas 1 and 2.

Chemerin toggled between two extremes; in a proximal conformation (replica 1), the chemerin N-terminus engaged the CCRL2 N-terminus within the first 500 ns (Figure 5A,D) supported by hydrophobic contacts (Y31_CCRL2_-D57_chem_; A38_CCRL2_ and L22_chem_), a hydrogen bond (Q34_CCRL2_–T23_chem_), and a stabilizing Y276_CCRL2_ (TM7)–V56_chem_ contact, consistent with an anchoring role near TM1 and the CCRL2 N-terminal (Supporting Information Figure S12A). In replica 2, this proximal binding mode was transient and was followed by partial dissociation (Figure 5B,D). In a distal conformation (replica 3), the chemerin N-terminus remained separated from CCRL2’s N-terminal region despite persistent ECL2−β1 contact (Figure 5C,D); here, the chemerin α2 helix lay near the extracellular TM4-TM5 surface and was stabilized by a π-cation interaction (F195_CCRL2_–R125_chem_), a D192_CCRL2_-R125_chem_ salt bridge, π–π (W196_CCRL2_–F156_chem_) and hydrophobic contacts involving F156_chem_ and V168/V169/W196 of CCRL2 (Supporting Information Figure S12B) with additional engagement of helix H2 of chemerin (specifically residues L136_chem_, Q139_chem_, and R140_chem_) with L189_CCRL2_, P190_CCRL2_, and A191_CCRL2_. Contact maps corroborated greater N-terminal proximity in replicas 1–2 (>5% N-terminal–N-terminal contacts) that in replica 3 (Supporting Information Figure S10A–C) with minor adjustments in the chemerin core relative to the CCRL2 helical bundle. In line with recent cryo-EM structure of the chemerin–CMKLR1–G_i1_ and chemerin–GPR1–G_i1_ complexes, distinct ligand orientations were revealed.^81^ In the chemerin–CMKLR1–G_i1_ complex, chemerin core adopts a perpendicular orientation relative to the membrane, whereas in the chemerin–GPR1–G_i1_ complex, it tilts approximately 45° toward ECL2.

Consistent with the GPR1 complex, our MD simulations indicate that chemerin predominantly adopts a tilted orientation toward the ECL2 of CCRL2. Across all simulations, ECL2-β1 interactions remained stable and chemerin exchanged “distal conformation” and a “proximal conformation”, that respectively disengage from, or engage with CCRL2’s extracellular and N-terminal regions (Figure 5A,B).

### Electrostatic Anchoring of Chemerin via Loop 3 and N-Terminal Domain of CCRL2

An additional interaction interface identified in our AA-MD contact map (Figure 4) involves chemerin loop 3 (located between β2 and β3; Supporting Information Figure S1C) and the N-terminal domain of CCRL2.

Contact analysis reveals interactions between CCRL2 residues L6, P8, E9, D10, and E11 and chemerin residues K71, K79, W82, R90, P91, G93, R94, and K95. These contacts are primarily electrostatic and feature several persistent salt bridges. Specifically, E9 of CCRL2 forms salt bridges with K71, K79, and K95 of chemerin, while E11 forms an additional salt bridge with K95. E9 also participates in hydrogen bonding with R94 and K95 of chemerin. Additional hydrogen bonds are observed between R90 of chemerin and N3 of CCRL2, as well as between T5 of chemerin and N92 of CCRL2. Although less frequent, vdW interactions are also detected. A prominent feature of this interface is the pronounced electrostatic complementarity between highly basic loop 3 of chemerin and the acidic N-terminal domain of CCRL2. This is illustrated by the electrostatic surface potential map (Figure 6), where the positively charged region of chemerin aligns with the negatively charged surface of CCRL2 (highlighted in the purple box in Figure 4).

**Figure 6:**
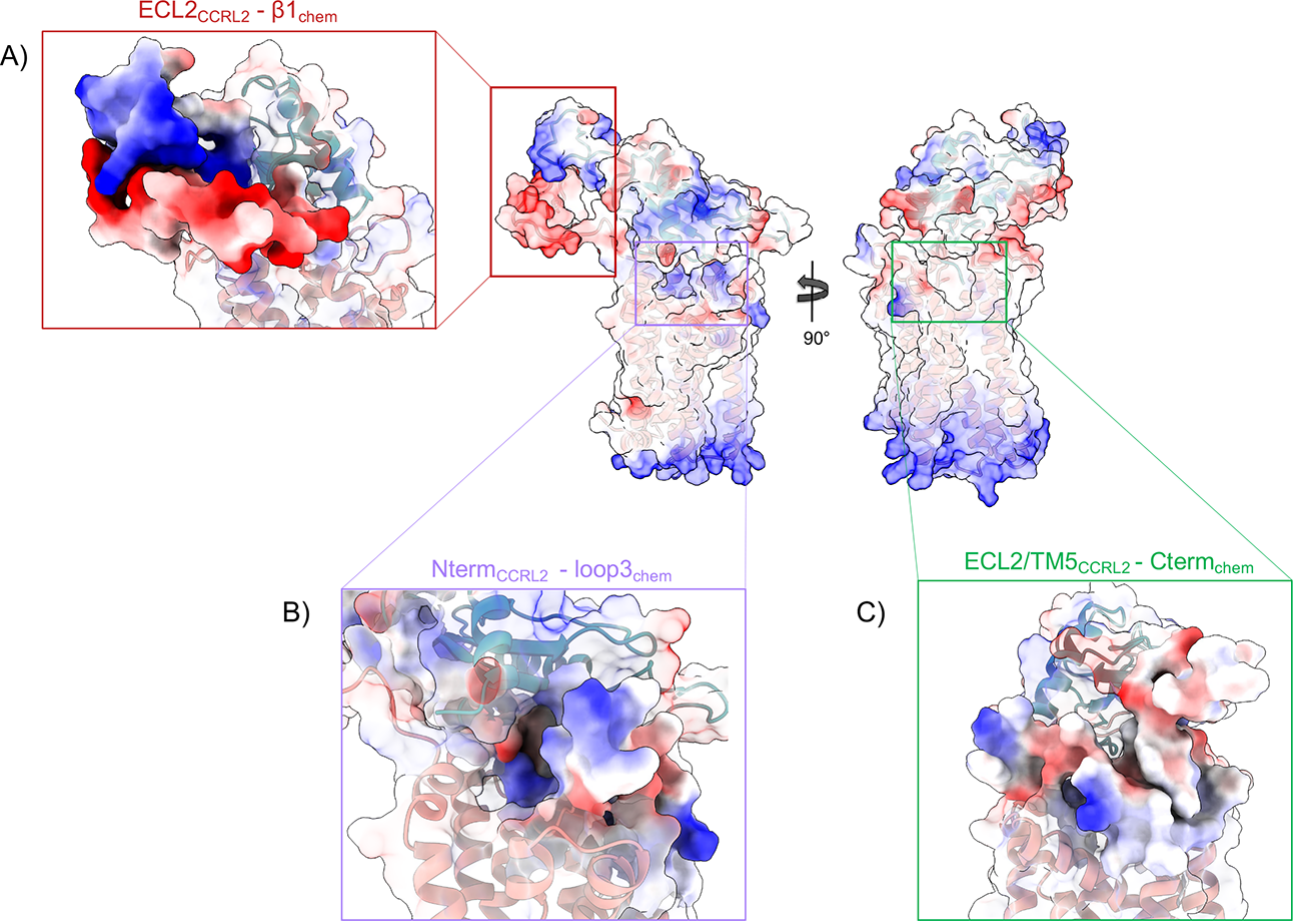
Electrostatic potential surfaces of the CCRL2–chemerin complex. Electrostatic surfaces for CCRL2 and chemerin are shown for the representative structure of the most populated cluster from replica 1. Positive potential is colored blue, neutral white, and negative red. Insets focus on key interaction regions highlighted in Figure 4: (A) ECL2_CCRL2_–β1_chem_, (B) N-term_CCRL2_–loop3_chem_, and (C) ECL2/TM5_CCRL2_–C-term_chem_.

This electrostatic complementarity closely resembles the classical chemokine recognition site 1 (CRS1), characterized by ionic interactions between basic residues on the chemokine and acidic residues on the receptor’s N-terminus and extracellular loops.^82^ Although CCRL2 binds chemerin in an orientation similar to that observed in the chemerin–GPR1 complex, its N-terminal engagement mode differs markedly from that of GPR1 and CMKLR1. In these latter receptors, the N-terminal domain stabilizes the complex through extensive β-sheet-mediated polar interactions with the chemerin globular core.^18,27^ In contrast, CCRL2 anchors chemerin binding by wrapping its N-terminal region around basic loop 3, forming a network of electrostatic interactions that firmly stabilize the ligand in place.

### Noncanonical C-Terminal Interactions between Chemerin and CCRL2’s ECL2/TM5 Highlights a Presentation-Competent, Nonsignaling Binding Mode

The third interaction region identified in the contact map (Figure 4) involves chemerin’s C-terminal domain, and to a lesser extent, its H2 helix. These segments primarily interact with extracellular loop 2 (ECL2) and the extracellular portion of transmembrane helix 5 (TM5) of CCRL2. Key CCRL2 residues involved in this interface include V168, V169, L189, F195, W196, and F199 that predominantly contact chemerin residues F148, F156, and F157. Among these, the W196_CCRL2_–F156_chem_ interaction was the most frequent (60%) (Figure 4). This interface is stabilized by multiple vdW and π–π stacking interactions.

Chemerin functions as a “reverse chemokine,” utilizing both its N-terminal core and flexible C-terminal loop to engage receptors, in contrasts to the classical chemokine-receptor model, where the N-terminal activates the receptor and the C-terminus docks at the receptor surface.^27^ In active receptor complexes such as CMKLR1 and GPR1, chemerin’s C-terminal region inserts deeply into the orthosteric binding pocket. Cryo-EM structures have shown that residue F156_chem_ anchors at the base of the ligand-binding pocket through hydrophobic interactions with a conserved tyrosine at position 6.51 (CMKLR1: Y276^6.51^; GPR1: Y262^6.51^, using Ballesteros–Weinstein numbering).^81^

By contrast, CCRL2 does not follow this canonical binding mode, consistent with its classification as a nonsignaling receptor. Rather than inserting into the transmembrane core, chemerin’s C-terminal domain remains exposed and solvent-accessible, maintaining peripheral interactions with ECL2 and TM5 throughout the simulations. Notably, this region remains unbound within the helical bundle, preserving its availability for potential engagement with signaling receptors such as CMKLR1.

This peculiar structural arrangement supports the proposed role of CCRL2 as a chemerin-presenting molecule rather than a signaling receptor. The lack of deep C-terminal engagement aligns with the hypothesis that CCRL2 functions as a reservoir or scaffold for chemerin, facilitating its presentation to signaling-competent receptors.

### Structural Modeling of the CCRL2–Chemerin–CMKLR1 Axis Reveals a Mechanism for Ligand Presentation and Receptor Activation

Given that CCRL2 functions as a nonsignaling receptor that binds chemerin and promotes its presentation to the signaling receptor CMKLR1, we investigated whether the spatial arrangement and interactions observed in our simulations support a structural model of the CCRL2–chemerin–CMKLR1 axis. A recent cryo-EM study^27^ demonstrated that chemerin engages CMKLR1 through five distinct interaction sites, grouped into two chemerin binding regions (CBRs): CBR1, encompassing interaction sites IS1 and IS3, and CBR2, comprising IS4 and IS5. This binding mode has been independently validated by complementary structural studies.^81^

Analysis of the CMKLR1 structure revealed a ligand-binding pocket within the transmembrane domain that accommodates the C-terminal region. Consistent with previous findings,^83^ full-length chemerin exhibits greater agonistic activity in activating CMKLR1 and GPR1 compared to the shorter C9 peptide, suggesting that the chemerin core contributes additional receptor contacts. Notably, interactions at CBR1 are stabilized through an extended hydrogen-bond network between chemerin’s β4 strand and the N-terminal β-strand of CMKLR1, forming a β-sheet-like interface critical for receptor docking. Meanwhile, residues 129–137 of chemerin (IS4 and IS5) further stabilize the interaction by anchoring the C-terminal domain in an S-shaped conformation within the orthosteric pocket (CBR2). Our simulations indicate that CCRL2 primarily engages in the N-terminal region of chemerin, including the β1 strand and loop3. While the C-terminal domain forms occasional contacts with CCRL2, it remains largely solvent-exposed and outside the transmembrane bundle. Based on these observations, we constructed a preliminary model of the full CCRL2–chemerin–CMKLR1 complex by integrating the cryo-EM structure of the CMKLR1–chemerin complex (PDB ID: 8ZJG) with a representative CCRL2–chemerin conformation from our all-atom MD simulations. Specifically, we selected a frame in which the β4 strand and C-terminal domain of chemerin were solvent-accessible and optimally oriented for CMKLR1 binding (Supporting Information Figure S19).

As illustrated in Figure 7, the resulting model places CCRL2 (light blue) and CMKLR1 (orange) on opposite sides of the remodeled chemerin (gray), which includes computationally reconstructed loops absent in the cryo-EM structure.

**Figure 7:**
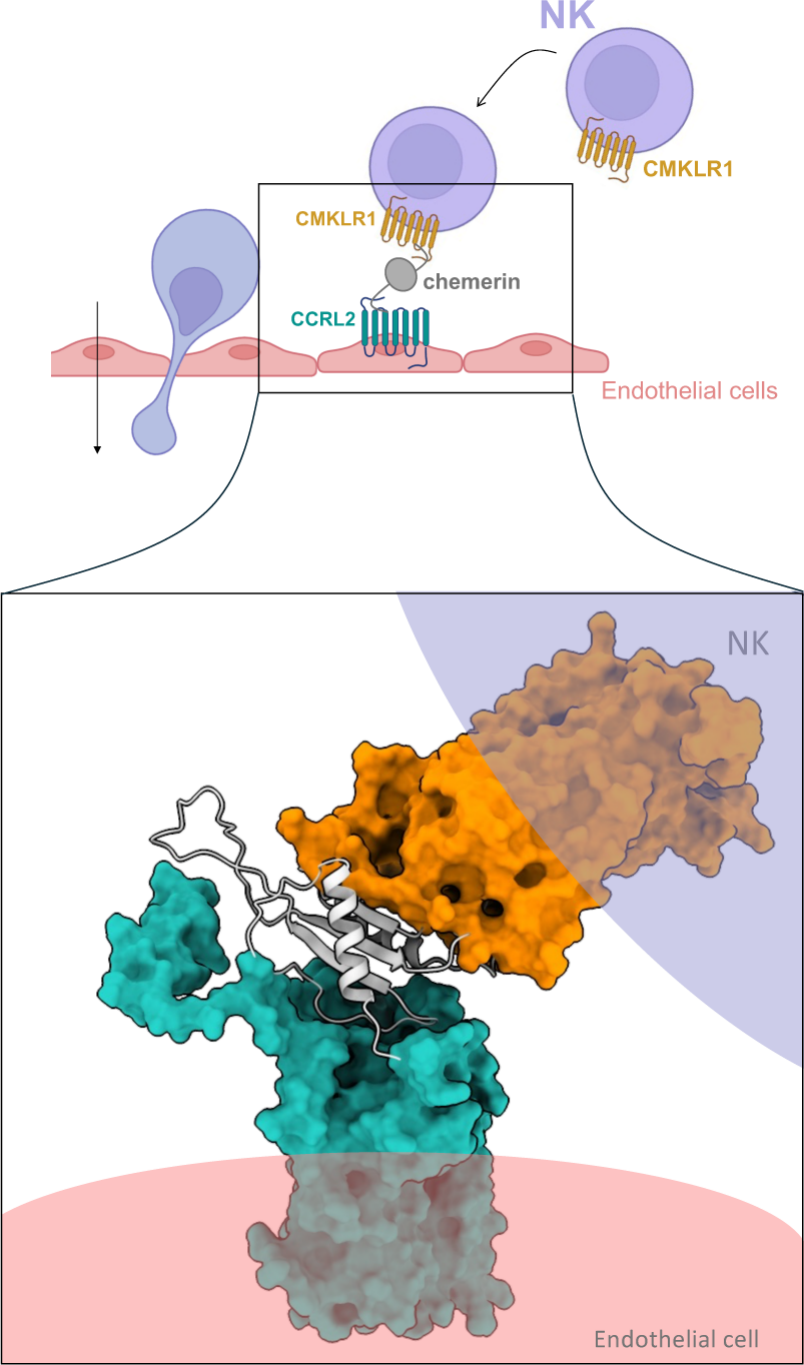
Structural model of the CCRL2–chemerin–CMKLR1 complex. CCRL2 (light blue) and CMKLR1 (orange) are positioned on either side of remodeled chemerin (gray), which incorporates reconstructed loops missing from the original cryo-EM structure. The model was generated using a template-based approach, aligning main-chain atoms of the cryo-EM resolved chemerin (PDB: 8ZJG) with the chemerin conformation in the CCRL2–chemerin complex from our simulations. This structural arrangement supports the feasibility of CCRL2-facilitated chemerin presentation to CMKLR1, highlighting a potential mechanism for leukocyte recruitment.

This hypothesized model, developed through our multiscale simulation pipeline, supports the notion that the CCRL2-bound chemerin can adopt a conformation compatible with CMKLR1 engagement. Further refinement, including EM and extended MD simulations, will be required to achieve a more realistic and dynamically stable representation of the CCRL2–chemerin–CMKLR1 axis.

### Integrative Studies of Pathogenic Missense Variants at the CCRL2–Chemerin–CMKLR1 Interaction Interface

We examined missense variants that could perturb CCRL2–chemerin and chemerin–CMKLR1 interactions. Variants in CCRL2, chemerin, and CMKLR1 were retrieved from the Genome Aggregation Database (gnomAD)^84^ and filtered by (i) AlphaMissense (AM) score and classification (likely pathogenic, ambiguous, likely benign)^68^ and (ii) localization to the protein–protein interface defined by contact heatmaps (Figure 4). AM thresholds were 0–0.33 (benign); 0.34–0.564 (ambiguous); and 0.565–1 (pathogenic), with corresponding color codes shown in Supporting Information Figure S13.

In CCRL2, likely pathogenic variants were primary located in the transmembrane helices, ECL2, ECL3, and the N-terminal region (Supporting Information Figures S13 and S14). Most variants at the interface hotspots were classified as ambiguous. In chemerin, likely pathogenic variants were enriched in β2−β4 strands and the C-terminal region (Supporting Information Figures S13 and S15), whereas CMKLR1 showed a higher concentration of predicted pathogenic variants in its extracellular loops (Supporting Information Figure S16).

To explore potential functional effects on CCRL2–chemerin binding, we analyzed representative cluster conformations from CG-MD simulations (Figure 4). Binding stability changes (ΔΔ*G*) were estimated with FoldX tool (version 5.1);^69^ positive ΔΔ*G* value indicates destabilization. Predictions were cross-validated using MutaBind2,^70^ DynaMut2,^71^ and DDMut-PPI,^72^ which yielded consistent trends (Supporting Information Table S4).

Several variants were predicted to directly influence the CCRL2–chemerin interface including P61S_chem_ and P172T_CCRL2_, P172L_CCRL2_ and F188I_CCRL2_, which affect interactions between CCRL2 ECL2 and the chemerin β1 strand (Supporting Information Table S2). Proline substitutions, such as P61_chem_, could alter β-sheet integrity via local flexibility changes and P172_CCRL2_ and F188_CCRL2_ substitutions may perturb π–π or cation–π interactions at interface. Additional variants were identified in CCRL2’s N-terminal region and chemerin’s loop3 (Supporting Information Table S2), where electrostatic contacts appear important (see Figure 6) including E11A_CCRL2_, E11G_CCRL2_ and R90S_chem_, W82G_chem_ (Supporting Information Table S2), any of which could weaken salt bridges or cation–π interactions. Y31N_CCRL2_ and Y31H_CCRL2_ (Figure 8A) may hinder the flat N-terminal conformation associated with stable receptor engagement (Figure 5E,F).

**Figure 8:**
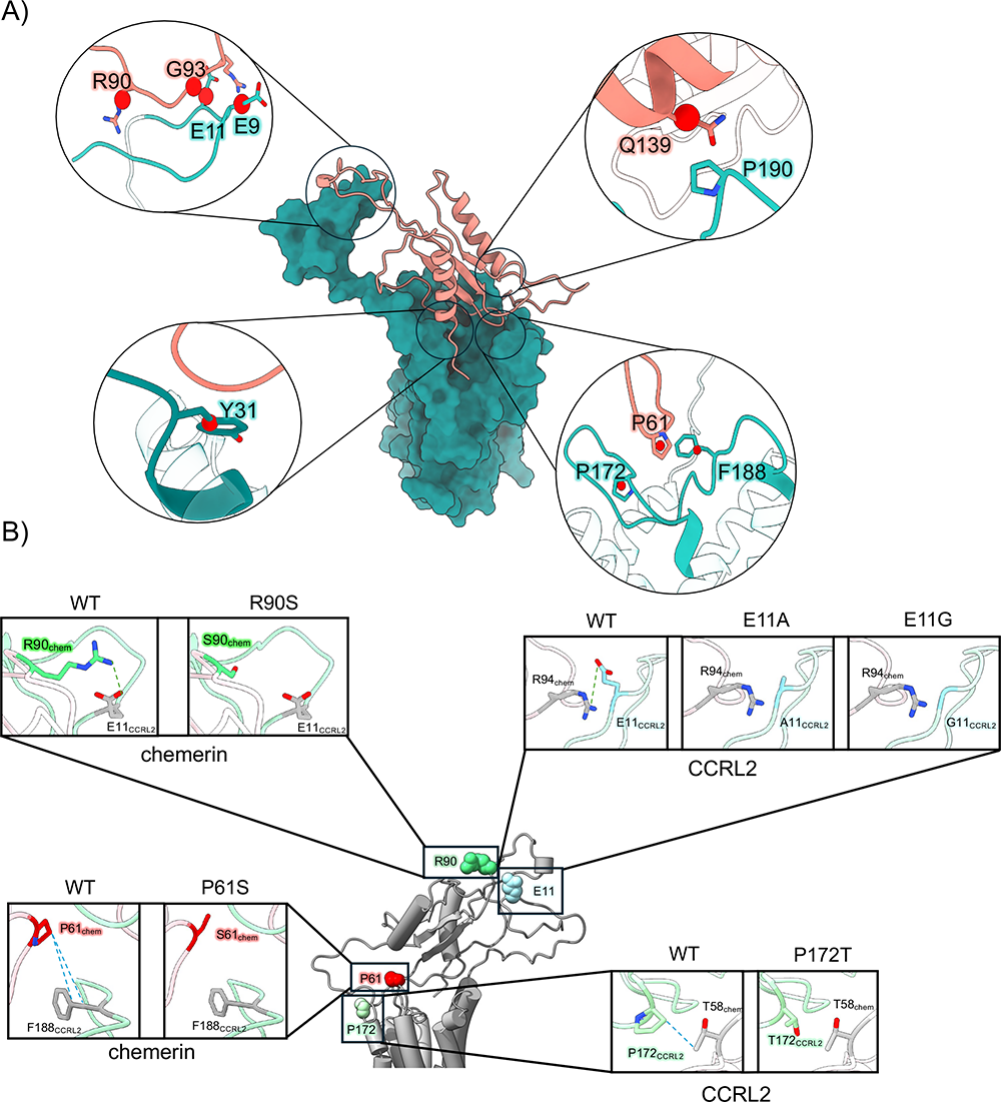
Mutation-induced structural perturbations at the CCRL2–chemerin interaction interface. (A) CCRL2 (light blue surface) and chemerin (light pink cartoon) with interface regions highlighted. Red spheres represent mutated residue centroids. (B) Close-up views show key missense variants: P61S_chem_ in loop2 (residues 60–63), R90S_chem_ in loop3 (residues 74–95), E11A_CCRL2_ in the N-terminal region (residues 1–31), and P172T_CCRL2_ in ECL2 (residues 166–196). Blue, yellow, and green dashed lines represent hydrogen bonds, hydrophobic interactions, and salt bridges, respectively. In particular, P61S_chem_ (ΔΔ*G* = 2.2 kcal/mol) may destabilize loop2 by replacing proline with serine in a flexible region. R90S_chem_ (ΔΔ*G* = 4.7 kcal/mol) may disrupt salt-bridge formation with E11A_CCRL2_, which forms multiple contacts with chemerin loop3. E11A_CCRL2_ (ΔΔ*G* = 4.7–4.9 kcal/mol) likely abolish these electrostatic interactions. P172T_CCRL2_ may interfere with π-stacking or cation–π interactions involving β1 and loop2 of chemerin.

Although Q139E_chem_ variant show a modest ΔΔ*G* (−0.5 kcal/mol), it could influence a proposed cation–π interaction with F188 (CCRL2) and thereby subtly shift the local electrostatic environment (Figure 8A). Five variants with comparatively higher predicted impact, P61S_chem_, R90S_chem_, E11A_CCRL2_, E11G_CCRL2_, and P172T_CCRL2_, are highlighted in Figure 8B.

Complex stability was further characterized using the Protein Contacts Atlas^76^ (Supporting Information Figure S17B,C, S18B and Table S5). Residue-interaction networks indicated hub-like positions with high centrality and low solvent accessibility for chemerin residues P61, K71, W82, R90, G93, and Q139 (Supporting Information Figure S17) and CCRL2 residues E9, E11, P172, and F188 (Supporting Information Figure S18), consistent with potential structural relevance.

Analysis of CMKLR1 suggested several ECL2 variants at the chemerin-binding interface, including R178W_CMKLR1_, R178Q_CMKLR1_, N191I_CMKLR1_, and N191S_CMKLR1_, could be disruptive (Supporting Information Table S3) with R178W_CMKLR1_ showing the largest predicted destabilization (FoldX ΔΔ*G* = 8.2 kcal/mol).

Collectively, these in silico results are consistent with the possibility that a subset of gnomAD-listed variants at the CCRL2–chemerin interface may reduce complex stability. While AM classifications and ΔΔ*G*-based estimates provide a coherent prioritization framework, most variants lack ClinVar annotations,^85^ likely reflecting low allele frequencies, and F188I_CCRL2_ was classified as of “uncertain significance”.^85^ Accordingly, the findings should be considered hypothesis-generating: targeted biochemical, biophysical, and genetic studies will be needed to determine whether the highlighted variants have consistent effects on receptor–ligand recognition and clarify any clinical implication.

## Conclusions

This study presents a comprehensive multiscale computational investigation of the CCRL2–chemerin interaction, revealing the structural and dynamic determinants underlying CCRL2’s noncanonical function as a chemerin-presenting receptor. In the absence of an experimentally solved CCRL2 structure, we employed AlphaFold2 to generate a reliable structural model. Although the noncanonical features of CCRL2 may be underrepresented in an AlphaFold-predicted fold constrained by canonical GPCR templates, our multiscale simulations consistently reproduced behaviors characteristic of atypical GPCRs, thus strengthening the solidity of our model. We also acknowledge the intrinsic limitations of conventional MD simulations, which, even with extended time scale and multiscale approaches, may not fully capture slow conformational transitions and low-energy states.^86–88^ Future efforts integrating enhanced sampling technique alongside experimental validation will be essential to refine our current model.

By integrating coarse-grained and all-atom MD simulations with pathogenic variant analyses, we demonstrated that CCRL2 engages chemerin primarily through its extracellular loop2 (ECL2) and N-terminal domain, while maintaining chemerin’s C-terminal region available for CMKLR1 binding. This organization supports a nonsignaling, presentation-competent interface that promotes leukocyte recruitment during inflammation. Structural mapping of naturally occurring CCRL2 missense variants further highlights potential molecular perturbations at the CCRL2 and chemerin–CMKLR1 interface that may contribute to immune dysregulation.

Although most of the reported variants are rare and remain poorly characterized clinically, our impact prediction will provide a starting point for future experimental and clinical investigation. Notably, CCRL2 variants, located outside the chemerin-binding interface, have associated with an increased risk of lung diseases.^89,90^ More broadly, CCRL2 is expressed mostly by barrier and myeloid cells, and it is able to regulate leukocytes migration.^6^ In fact, when expressed by endothelial cells, CCRL2 can act as a chemerin-presenting molecule, thus locally concentrating chemerin to recruit on site ChemerinR1^+^ innate immune cells like NK (Natural Killers) or dendritic cells.^10,91,92^ Therefore, as CCRL2 expression by endothelial cells is increased upon inflammatory stimuli,^93^ its role as an innate immune cell homing receptor could be harnessed to enhance immune surveillance in cancer or infection diseases. Moreover, considering the plethora of roles played by chemerin in various pathophysiological contexts,^6^ the ability to modulate CCRL2–chemerin interactions represents an attractive avenue for therapeutic intervention.

Overall, our findings provide mechanistic insight into CCRL2’s atypical receptor function, underscore its therapeutic potential in inflammatory pathologies, and highlight the strength of integrative, simulation-based strategies in elucidating noncanonical GPCR-ligand interactions.

## Supplementary Material



## Data Availability

All input files necessary to reproduce the molecular dynamics simulations and analysis scripts are deposited as zipped files in the Supporting Information.

## Notes

Structural models and domain architecture of CCRL2 and chemerin; rationale for *CG-binding_feasibility_simulations* extension; time-lag cross-correlation analysis of chemerin contacts with CCRL2_CRS1 and CCRL2_CRS2 across individual CG-MD simulations; RMSD analysis of CCRL2’s N-terminal domain and ECL2 in bound vs unbound states; representative CCRL2–chemerin complex conformations used in *CG-stable_binding_simulations*; RMSD analysis of *CG-binding_feasibility* and *CG-stable_binding simulations*; contact maps and PCA of CCRL2–chemerin interactions across three simulated conformations; backbone RMSD distributions across simulation types and structural regions; residue-wise RMSF profiles for CCRL2 and chemerin; heatmaps of CCRL2–chemerin contact frequencies across MD replicas; CloNe clustering of CCRL2–chemerin complexes; interaction maps of CCRL2–chemerin complexes from dominant clusters; structural mapping of CCRL2 and chemerin with AlphaMissense pathogenicity; regional pathogenicity mapping of CCRL2 residues; pathogenicity mapping for chemerin; pathogenicity mapping for CMKLR1 residues; network analysis of chemerin residues destabilizing the complex; network analysis of CCRL2 residues destabilizing the complex; final CCRL2–chemerin–CMKLR1 complex model integrating cryo-EM and MD data; overview of the 26 coarse-grained CCRL2–chemerin simulations; missense variant summary for CCRL2 and chemerin; missense variant summary for CMKLR1 and chemerin; ΔΔ*G*-based mutation impact on CCRL2–chemerin binding stability; and protein contact atlas analysis of CCRL2 and chemerin (PDF)
