# Supplementary material for: Multiscale Computational Dissection of CCRL2-Mediated Chemerin Presentation
Source: J Chem Inf Model. 2025 Dec 11;65(24):13400–19. doi: 10.1021/acs.jcim.5c01871 (PMC12728923; doi:10.1021/acs.jcim.5c01871)
Supplement: Supplementary file 1 [file ci5c01871_si_001.pdf]

Supporting Information for

Multiscale Computational Dissection of CCRL2-Mediated Chemerin Presentation

Arianna Migliorini[1], Samuele Di Cristofano[1], Klevia Dishnica[2,3], Alessandro Marchetto[4,5], Rui Pedro Ribeiro[3], Mattia Laffranchi[1], Elena Cerioni[1], Francesco Quilli[1], Eleonora Bonanni[1], Alejandro Giorgetti[3], Giulia Rossetti[4,6], Silvano Sozzani[1], Tiziana Borsello[787] and Domenico Raimondo[1,]*

[1]Department of Molecular Medicine, Laboratory Affiliated to Istituto Pasteur Italia - Fondazione Cenci Bolognetti, Sapienza University of Rome, Rome 00161, Italy

[2]Department of Chemistry, Bioscience and Environmental Engineering, Faculty of Science and Technology, University of Stavanger, Kristine Bonnevies vei 22, 4021 Stavanger, Norway.

[3]Department of Biotechnology, University of Verona, Strada le Grazie 15, 37134 Verona, Italy.

[4]Computational Biomedicine, Forschungszentrum Jülich, Wilhelm-Johnen-Straße, 52428 Jülich, Germany.

[5]Department of Biology, Faculty of Mathematics, Computer Science and Natural Sciences, RWTH Aachen University, Templergraben 59 D-52062 Aachen, Germany.

[6]Jülich Supercomputing Center, Forschungszentrum Jülich, Wilhelm-Johnen-Straße, 52428 Jülich, Germany, Department of Neurology, University Hospital Aachen, RWTH Aachen University, Pauwelsstraße 30, 52074 Aachen.

[7]Department of Pharmacological and Biomolecular Sciences, University of Milan, Via Balzaretti 9, 20133 Milan, Italy.

[8]Department of Neuroscience, Mario Negri Institute of Pharmacological Research IRCCS, Via Mario Negri 2, 20156, Milan, Italy.

*Corresponding author: Domenico Raimondo, Department of Molecular Medicine, Laboratory Affiliated to Istituto Pasteur Italia - Fondazione Cenci Bolognetti, Sapienza University of Rome, Rome 00161, Italy.

Email: domenico.raimondo@uniroma1.it

Table of Contents

Figure S1. Structural models and domain architecture of CCRL2 and chemerin

Figure S2. Rationale for CG-binding feasibility dynamics extension

Figure S3. Time-lag cross-correlation analysis of chemerin contacts with CCRL2_CRS1 and CCRL2_CRS2 across individual CG-MD simulations

Figure S4. RMSD analysis of CCRL2's N-terminal domain and ECL2 in bound vs. unbound states

Figure S5. Representative CCRL2–chemerin complex conformations used in CG-stable binding simulations

Figure S6. RMSD analysis of CG-binding_feasibility and CG-stable_binding simulations

Figure S7. Contact maps and PCA of CCRL2–chemerin interactions across three simulated conformations

Figure S8. Backbone RMSD distributions across simulation types and structural regions

Figure S9. Residue-wise RMSF profiles for CCRL2 and chemerin

Figure S10. Heatmaps of CCRL2–chemerin contact frequencies across MD replicas

Figure S11: CloNe clustering of CCRL2–chemerin complexes

Figure S12. Interaction maps of CCRL2–chemerin complexes from dominant clusters

Figure S13. Structural mapping of CCRL2 and chemerin with AlphaMissense pathogenicity

Figure S14. Regional pathogenicity mapping of CCRL2 residues

Figure S15. Pathogenicity mapping for chemerin residues

Figure S16. Pathogenicity mapping for CMKLR1 residues

Figure S17. Network analysis of chemerin residues destabilizing the complex

Figure S18. Network analysis of CCRL2 residues destabilizing the complex

Figure S19. Final CCRL2–chemerin–CMKLR1 complex model integrating cryo-EM and MD data

Table S1. Overview of the 26 coarse-grained CCRL2–chemerin simulations

Table S2. Missense variant summary for CCRL2 and chemerin

Table S3. Missense variant summary for CMKLR1 and chemerin

Table S4. ΔΔG-based mutation impact on CCRL2–chemerin binding stability

Table S5. Protein Contacts Atlas analysis of CCRL2 and chemerin

Supplementary Figures

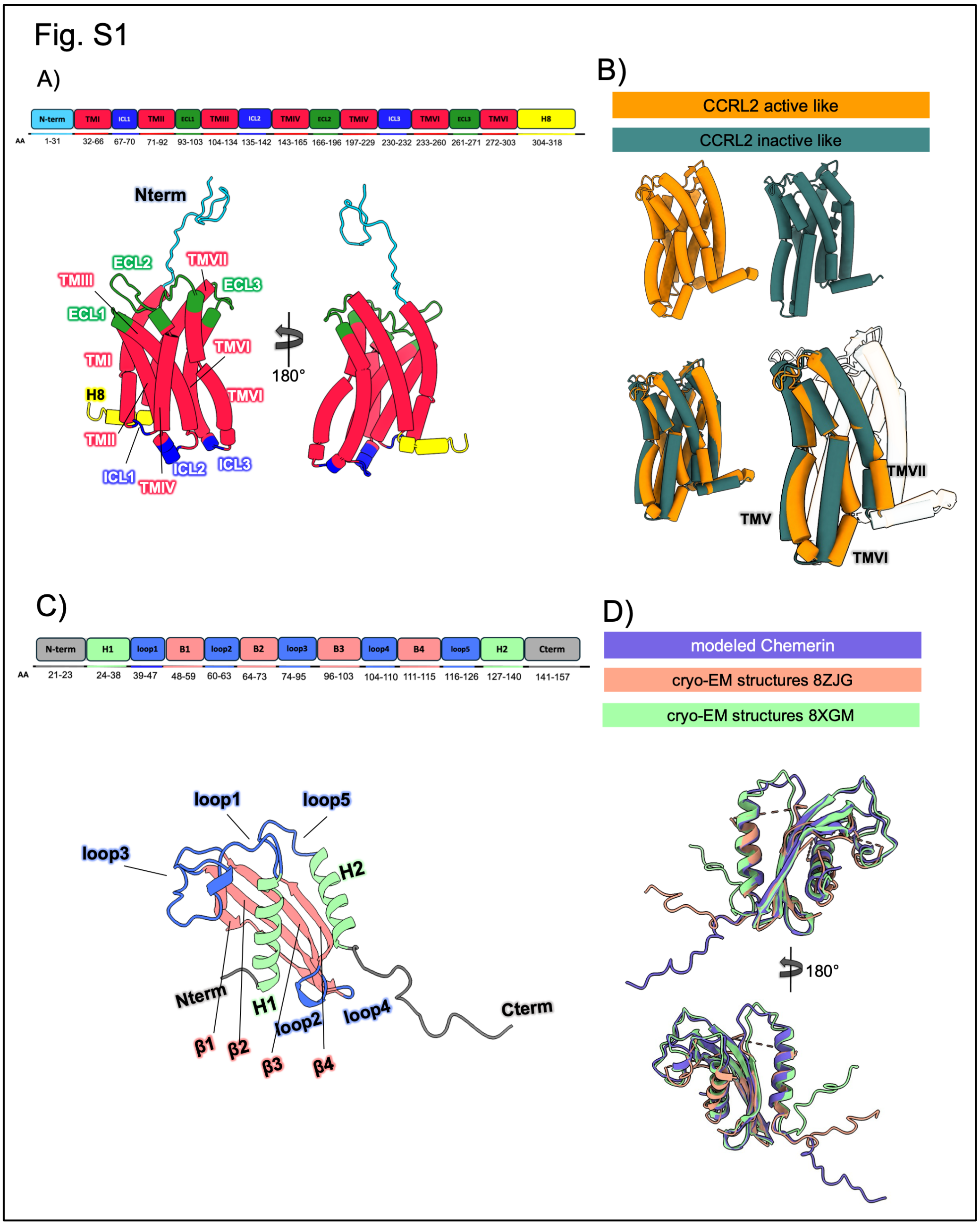

Figure S1. Structural models and domain architecture of CCRL2 and chemerin, aligned with experimental structures. (A) Schematic representation of CCRL2 showing terminal regions, transmembrane domains, and intra/extracellular loops, represented with domain-colored surfaces and a cartoon model. (B) Structural models of CCRL2 in predicted active (orange) and inactive (teal) conformations. Cα superposition reveals distinct conformational shifts: outward displacement of TMV and TMVI, and inward movement of TMVII in the active state. (C) Schematic of chemerin with terminal regions, β-strands, and loop segments colored by domain. Cartoon representations highlight loop1 (H1–β1), loop2 (β1–β2), loop3 (β2–β3), loop4 (β3–β4), and loop5 (β4–H2). Structural elements are shown with helices in light green, β-strands in pink, loops in light blue, and termini in grey. (D) Cα superposition of the in-house chemerin model (purple) with cryo-EM structures PDB:8ZJG (coral) and PDB:8XGM (green). The model aligns closely with 8XGM, with a global RMSD of 0.01 nm (excluding termini), indicating high structural fidelity.

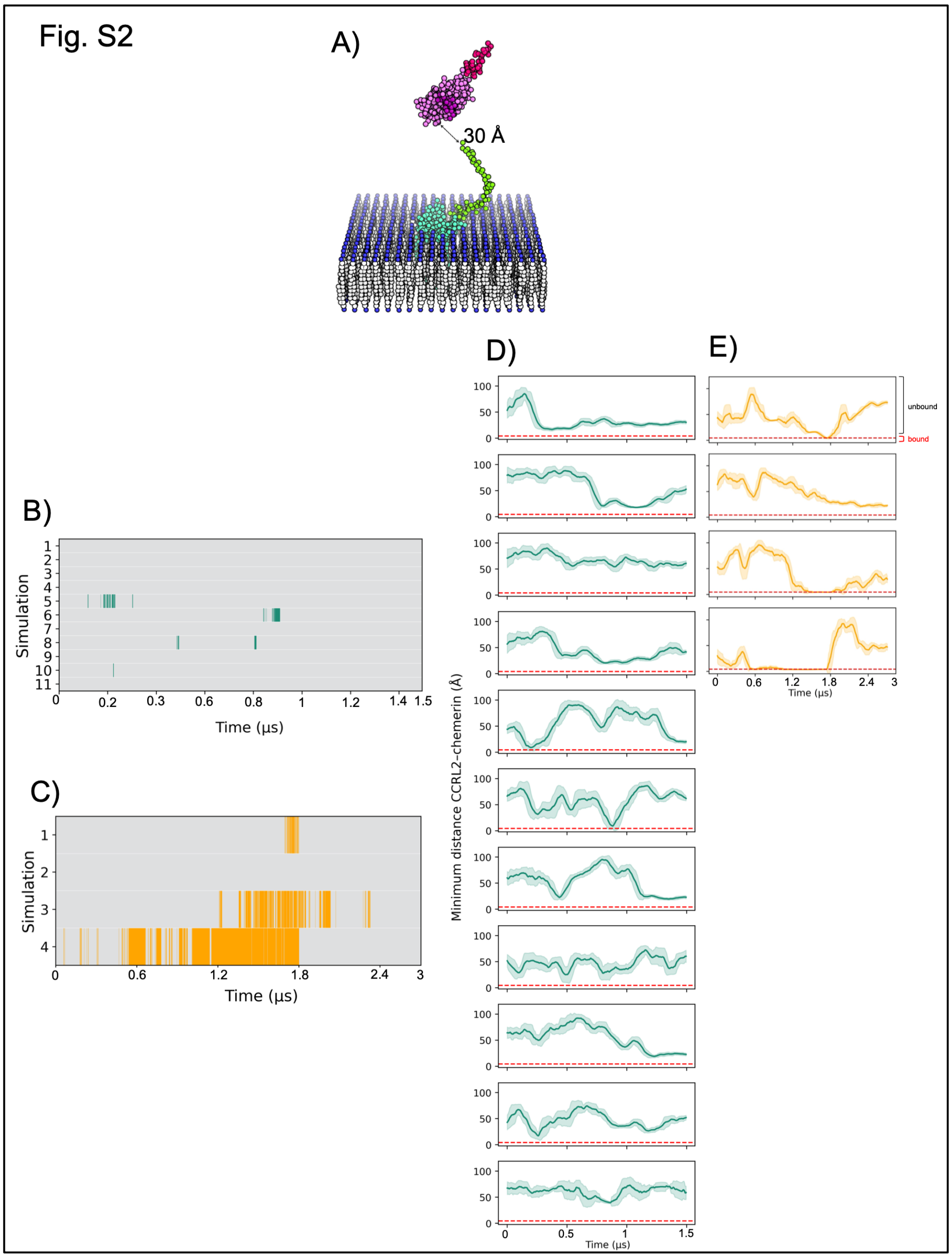

Figure S2. Rationale for CG-binding feasibility dynamics extension. System setup for coarse-grained binding feasibility simulations of CCRL2 and chemerin (A). Chemerin (pink) was placed 30 Å from the N-terminal residue of CCRL2 (green) to avoid initial bias. CCRL2 is shown in light blue, with phosphatidylcholine molecules in grey and blue. On- and off-bound states between CCRL2 and chemerin over 1.5 µs (B) and 3 µs (C) for the eleven and four trajectories, respectively, that were not extended for further analysis. Each frame is color-coded according to the minimum interatomic distance between the two proteins: gray indicates the unbound state (distance > 5 Å), green highlights ligand-bound frames in trajectories terminated at 1.5 µs, and orange marks bound frames in trajectories extended to 3 µs. Panels D–E report the minimum distance between any atom of CCRL2 and any atom of chemerin across the entire simulated time for trajectories stopped at 1.5 µs (green, D) and 3 µs (orange, E). The red line indicates the 5 Å threshold defining the bound regime. This representation provides additional insight into the overall distance fluctuations and spatial exploration of chemerin relative to CCRL2 when the two proteins are not in contact. As summarized in Table S1, trajectories stopped at 1.5 µs show no or minimal contact events, those stopped at 3 µs exhibit transient interactions.

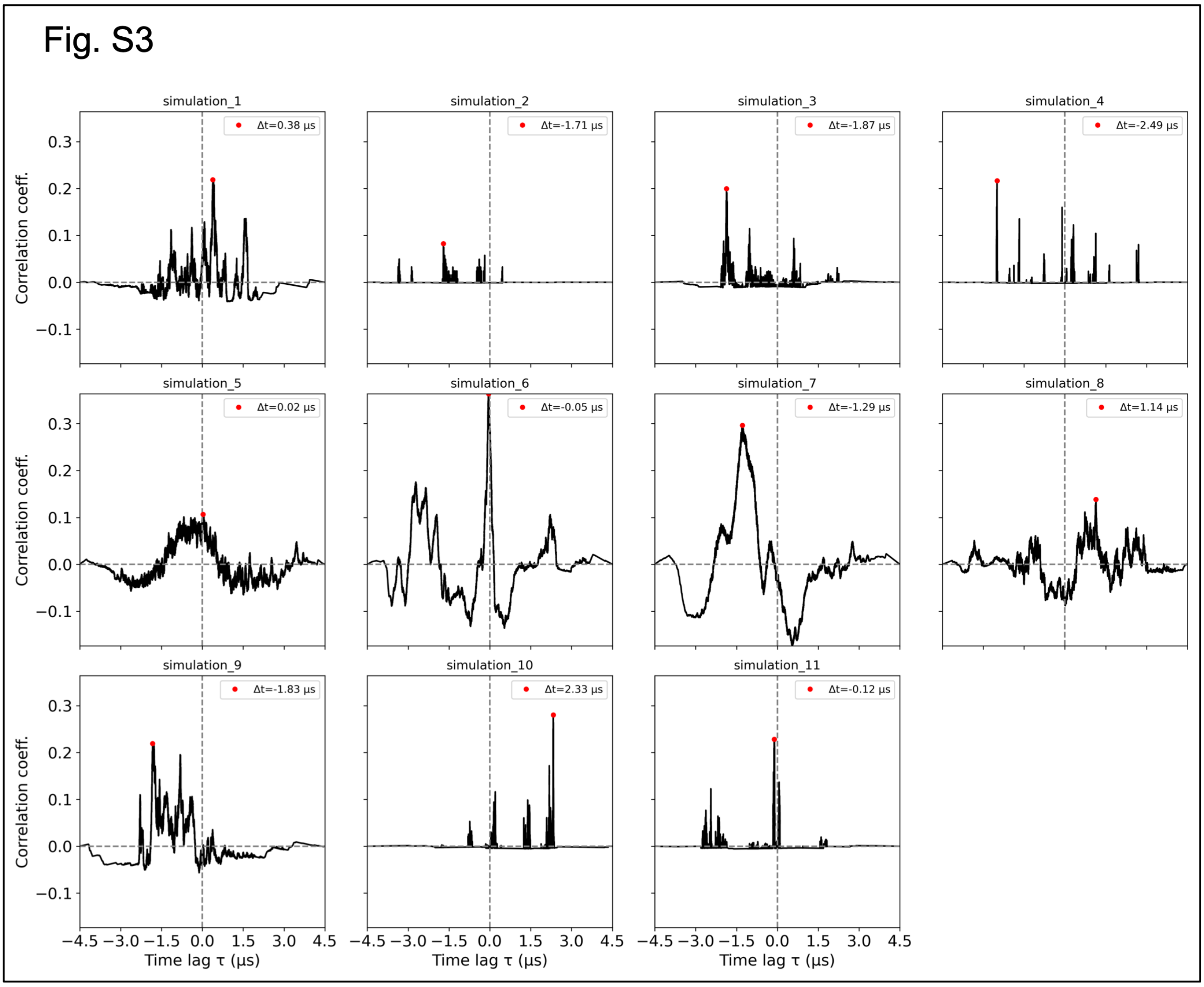

Figure S3. Time-lag cross-correlation analysis of chemerin contacts with CCRL2_CRS1 and CCRL2_CRS2 across individual *CG-binding_feasibility_simulations*. Each panel shows the cross-correlation coefficient as a function of time lag (τ, in μs). A negative lag at the maximum correlation (red dot) indicates that CRS1 engagement precedes CRS2, while a positive lag indicates the opposite. The Δt values report the lag at the correlation peak for each simulation.

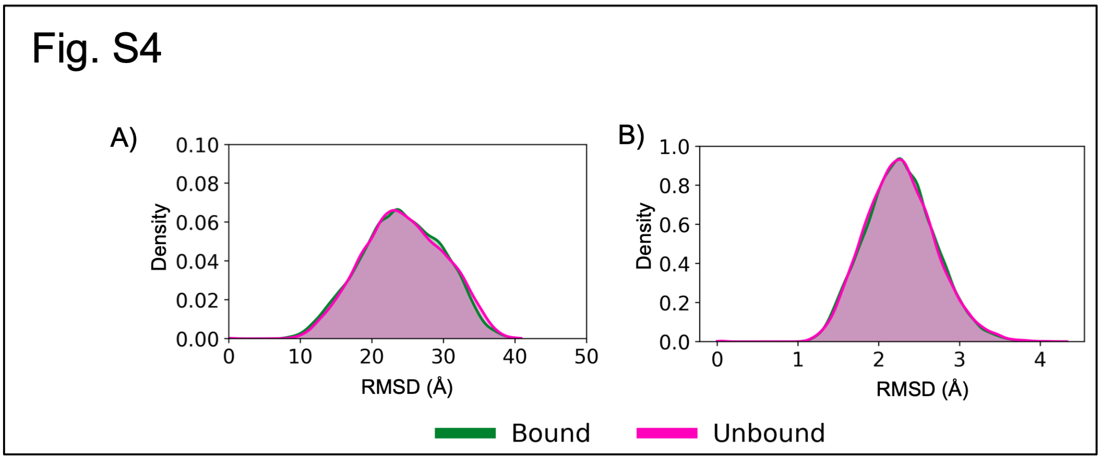

Figure S4. RMSD analysis of CCRL2's N-terminal domain and ECL2 in bound vs. unbound states. (A) RMSD distributions of the N-terminal domain (residues 1–31) show minor but significant differences between bound (green) and unbound (pink) frames (Kolmogorov-Smirnov test: D = 0.0186, p = 0.0004), suggesting slight binding-induced flexibility. (B) ECL2 RMSD distributions reveal no significant difference (D = 0.0095, p = 0.2121), indicating no stabilization upon chemerin binding.

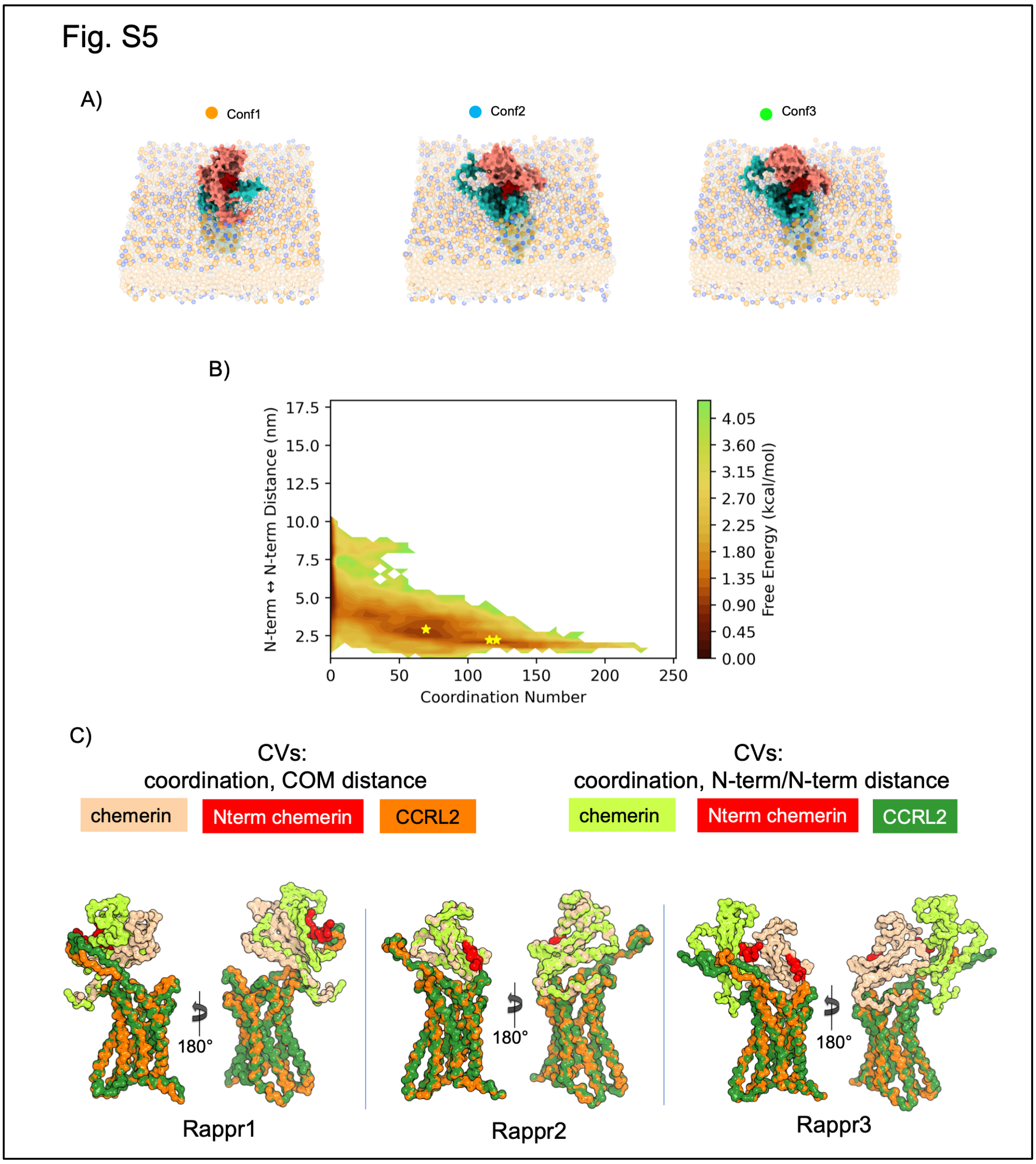

Figure S5. Representative CCRL2–chemerin complex conformations used in CG-stable binding simulations. (A) Structures from the three most populated clusters (orange, blue, green dots in Fig. 2A) are shown embedded in a transparent POPC membrane. CCRL2 is in light blue, chemerin in light pink, and its N-terminal domain in dark red. In Conf1, chemerin's N-terminal domain binds CCRL2's N-terminal region; in Conf2 and Conf3, it interacts with ECL2. (B) Free energy landscape (FEL) of the CCRL2–chemerin complex, calculated from 11 simulations using the coordination number and the N-terminal–to–N-terminal distance (orientation-dependent CV) as collective variables. The lowest energy regions (dark brown) correspond to conformations characterized by short N-terminal separation and high coordination, whereas higher free-energy regions are depicted from orange to green. The three lowest minima are indicated with yellow stars. (C) Superposition of representative structures from the most populated clusters corresponding to the lowest-energy basins of the FEL. Structures derived from the FEL computed with COM distance and coordination (CCRL2 in orange, chemerin in light brown), corresponding to Conf1, Conf2, and Conf3 in panel A and used as starting points for subsequent coarse-grained simulations, are compared with those obtained from the FEL computed with N-terminal–to–N-terminal distance and coordination (orientation-sensitive minima; CCRL2 in dark green, chemerin in light green), corresponding to the three yellow stars in panel B. In both cases, the N-terminal of chemerin is highlighted in red.

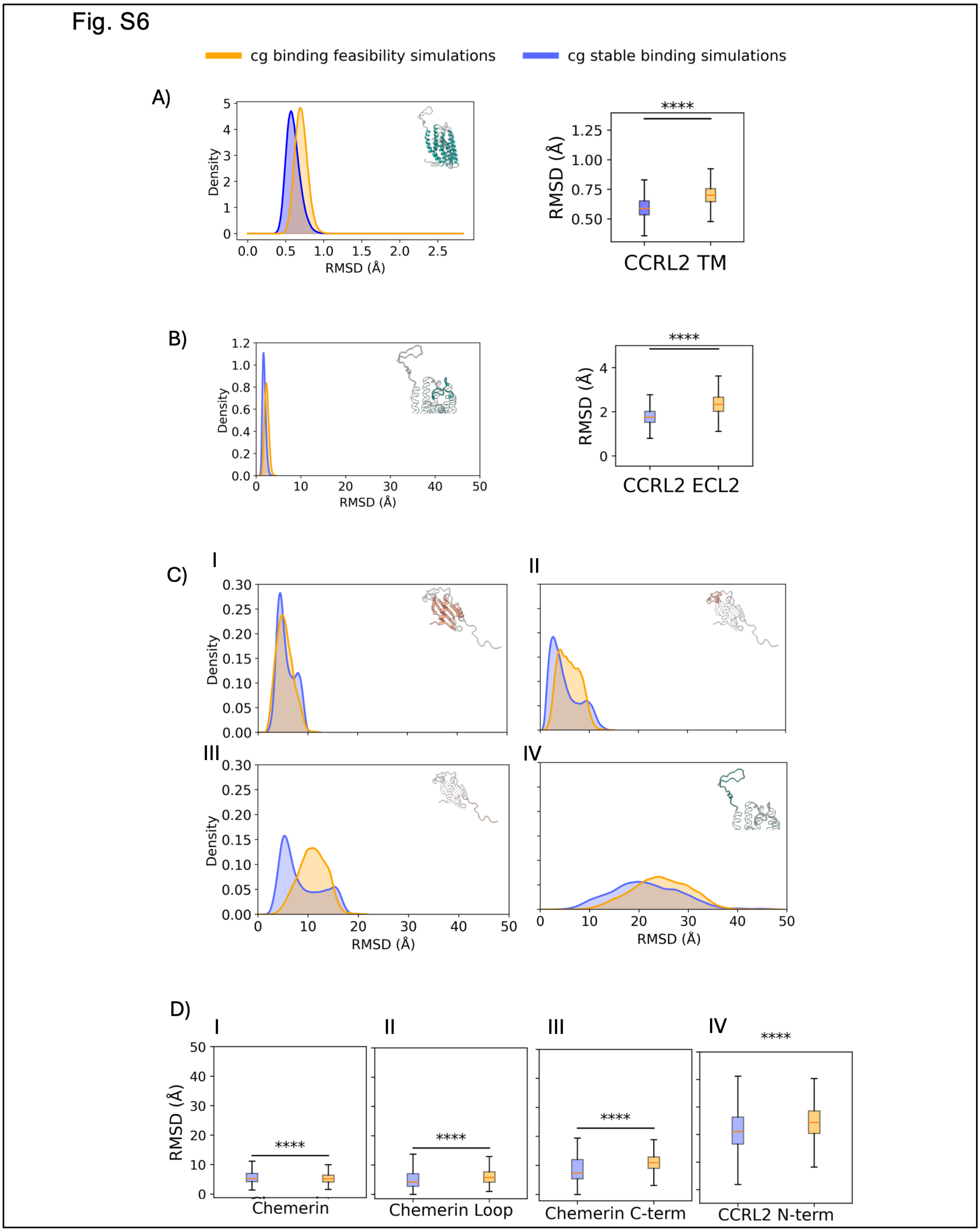

Figure S6. RMSD analysis of CG-binding feasibility and CG-stable binding simulations. Probability density distributions (A, B, left panels) and box plots (A,B, right panels; C) of backbone RMSDs are shown for all frames of the CG-binding feasibility simulations (blue) and CG-stable binding simulations (orange). (A) Internal fluctuations of the CCRL2 transmembrane (TM) domain. (B) Fluctuations of the CCRL2 ECL2 relative to the TM domain. (C) (I) Internal fluctuations of chemerin; (II) fluctuations of chemerin loop 2; (III) fluctuations of the chemerin C-terminal region; (IV) fluctuations of the CCRL2 N-terminal residues relative to the TM domain.

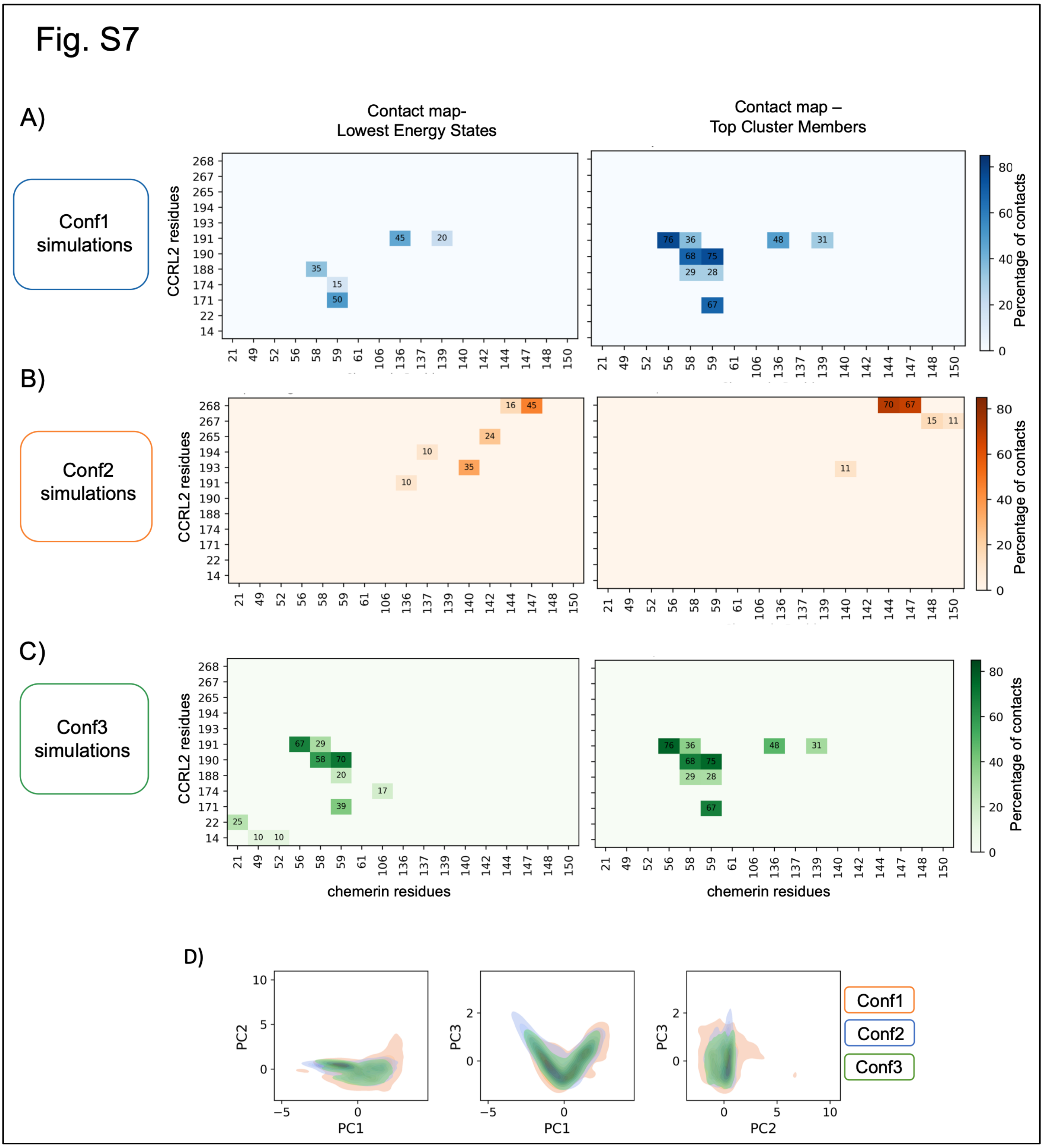

Figure S7. Contact maps and PCA of CCRL2–chemerin interactions across three simulated conformations. Maps for Conf1 (orange, A), Conf2 (blue, B), and Conf3 (green, C) show contacts persisting >10% of the simulation. The strongest contact (85%) occurs between chemerin residue 136 and CCRL2 residue 191. (D) Principal component analysis (PCA) of global descriptors (center-of-mass distance, intermolecular contacts, orientation angle) from the full trajectories of Conf1–3. Left: 3D PCA density plot showing the distribution of conformations in the first three principal components (PC1–PC3). Right: 2D contour projections of the same PCA, highlighting the pairwise distributions along PC1–PC2, PC1–PC3, and PC2–PC3.

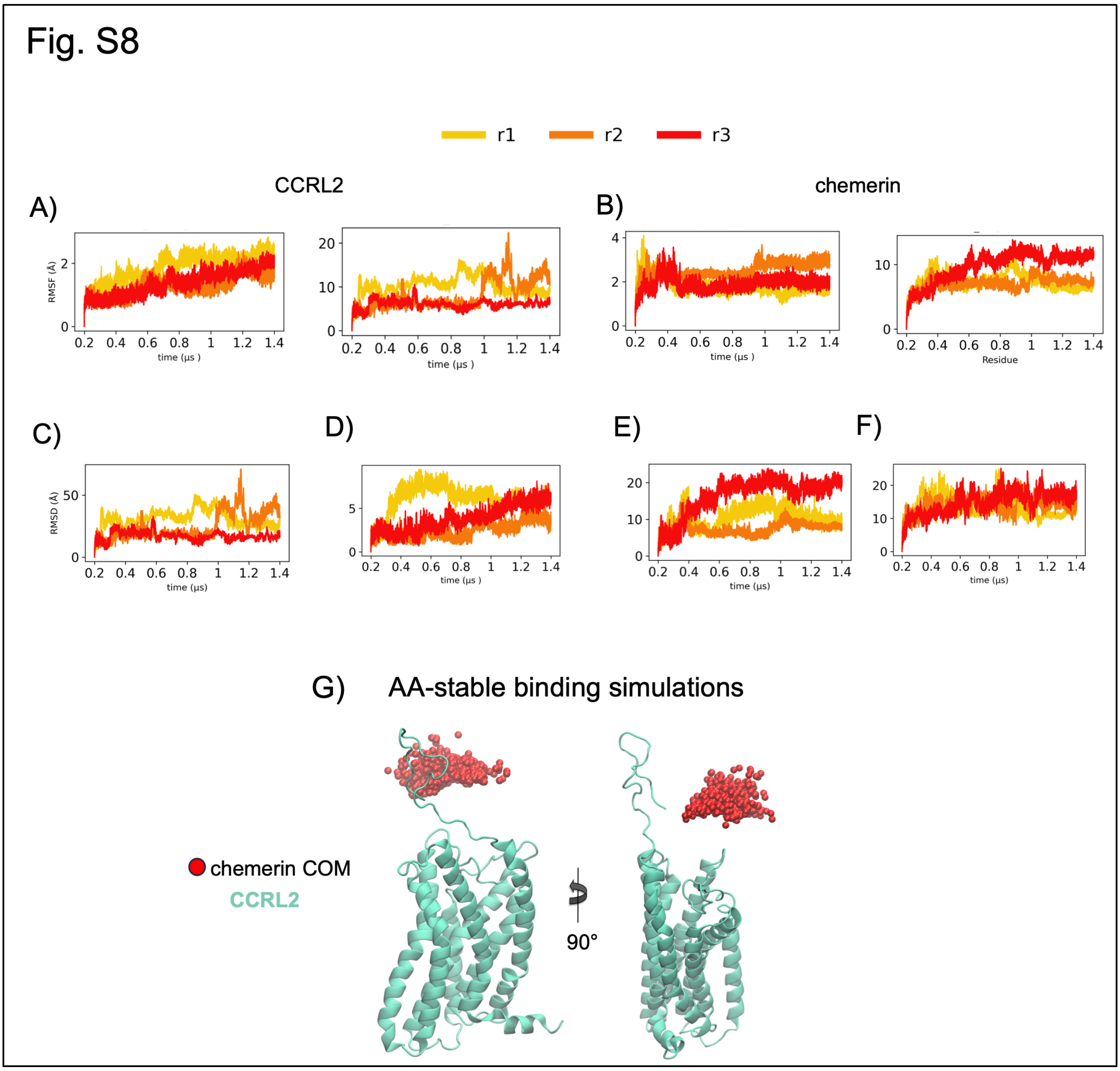

Figure S8. Backbone RMSD distributions across simulation types and structural regions. Time evolution for three AA-stable replicas (r1–yellow, r2–orange, r3–red) is presented. (A) Left: CCRL2 transmembrane Cα RMSD vs. itself. Right: CCRL2 RMSD vs. TM Cα atoms. (B) Left: chemerin secondary structure RMSD vs. itself. Right: chemerin RMSD vs. secondary structure Cα atoms. (C–F) RMSD of key domains relative to transmembrane or secondary elements, including N-terminal (C) and ECL2 (D) of CCRL2, and loop2 (E) and C-terminal (F) regions of chemerin. (G) Structural representation of CCRL2, shown as a light blue surface, with the COM positions of the chemerin core (excluding the C-terminal domain) represented as red dots periodically extracted from AA-stable binding simulation MD trajectories.

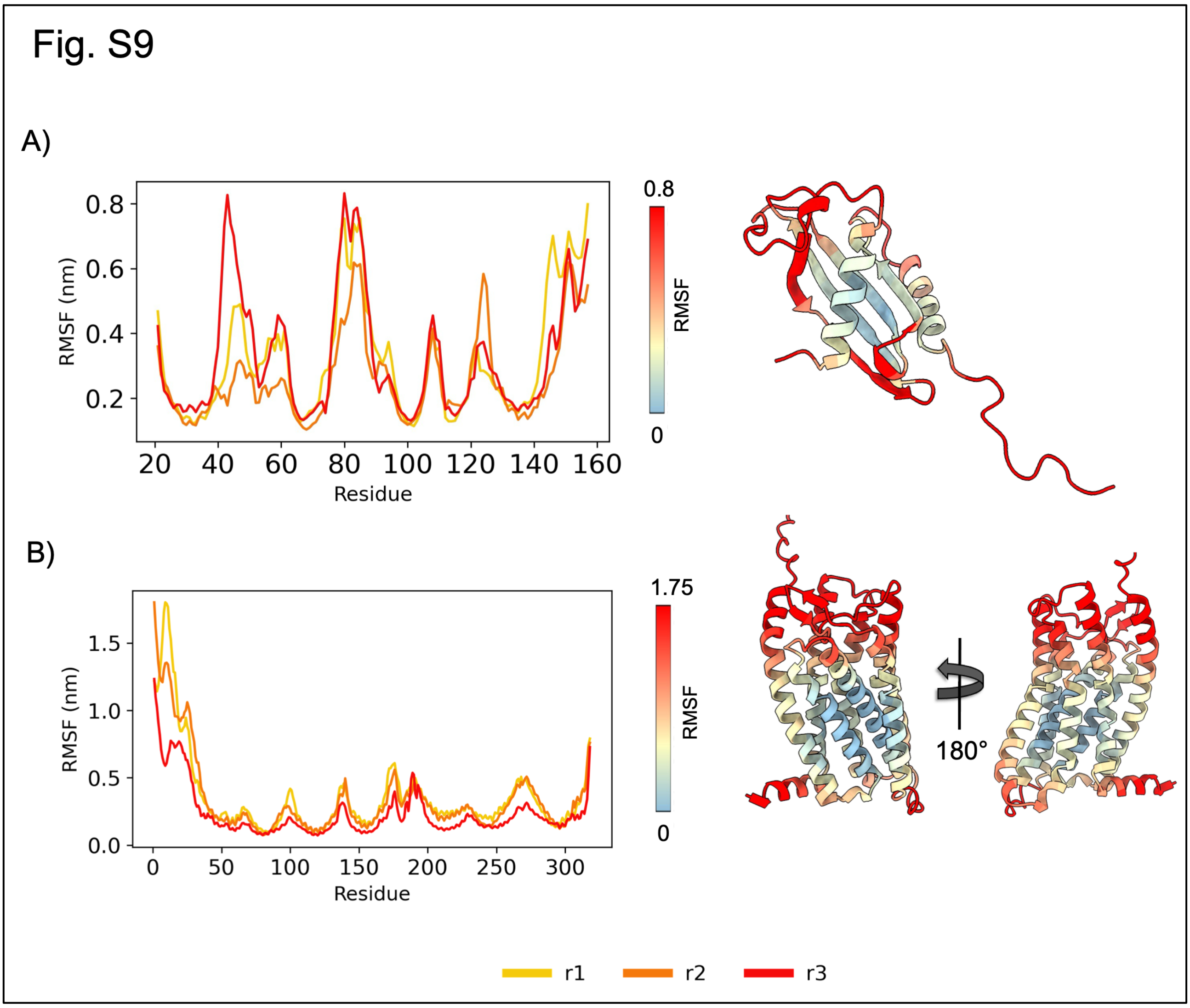

Figure S9. Residue-wise RMSF profiles for CCRL2 and chemerin. (A–B) Per-residue RMSF line plots for CCRL2 and chemerin are shown for three replicas (yellow, orange, red). RMSF values are mapped onto 3D structures using a cyan-to-red gradient. Flexible regions include chemerin loops 1–3 and CCRL2's N-terminal, TM1 (first 10 residues), TM5, and TM6.

Fig. S10

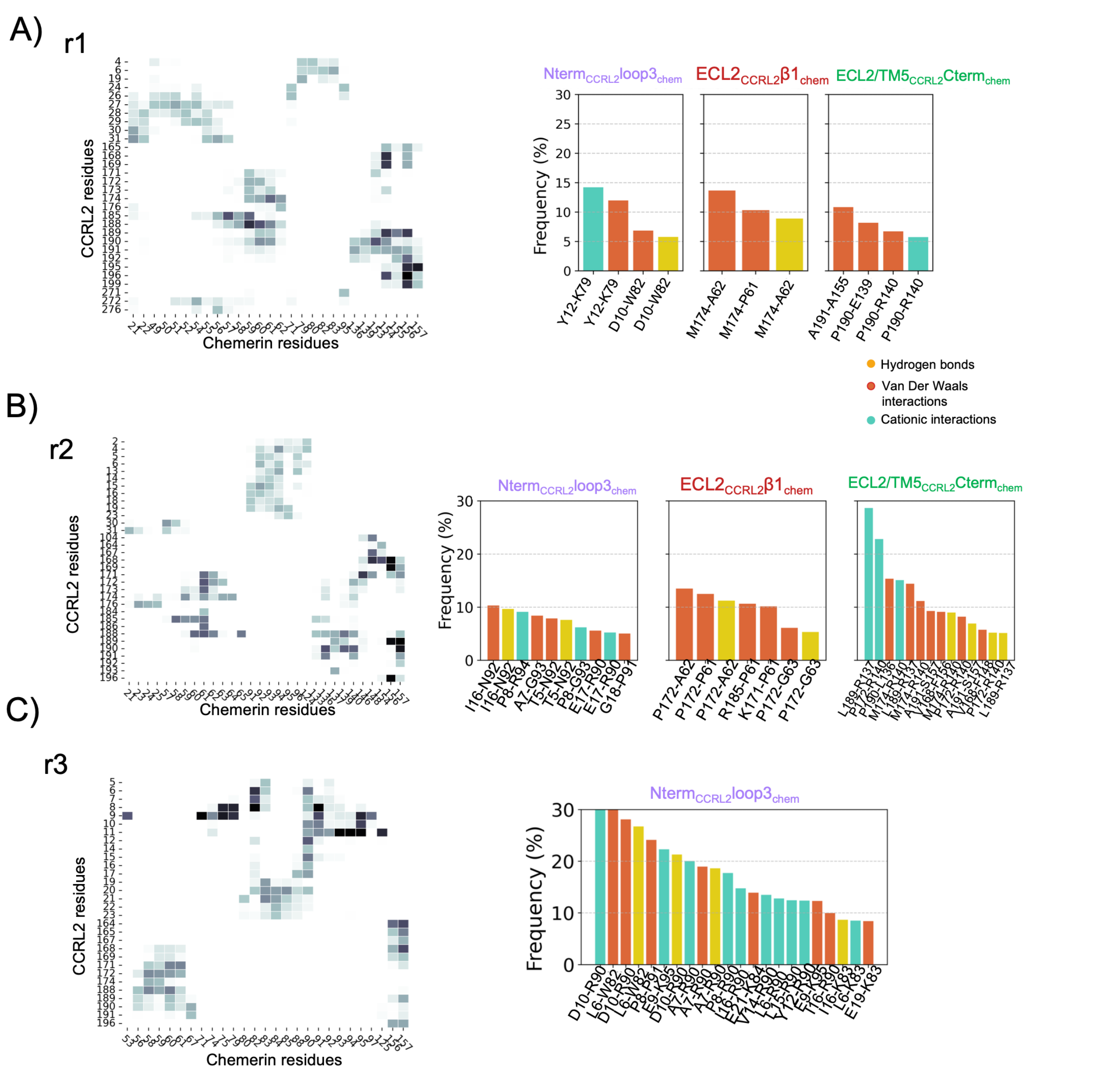

Figure S10. Heatmaps of CCRL2–chemerin contact frequencies across MD replicas. Contact maps show residue interactions persisting >5% of the time over 1.5 µs simulations. Panels A–C represent replicas 1–3, with darker gray indicating higher contact frequency. To the right of each panel, bar plots summarize the chemical interactions established during the dynamics within the regions highlighted in Fig. 4. Only contacts with frequency >5% are shown. Interaction types are color-coded: van der Waals (red), hydrogen-bond donor (yellow), and cationic (light blue).

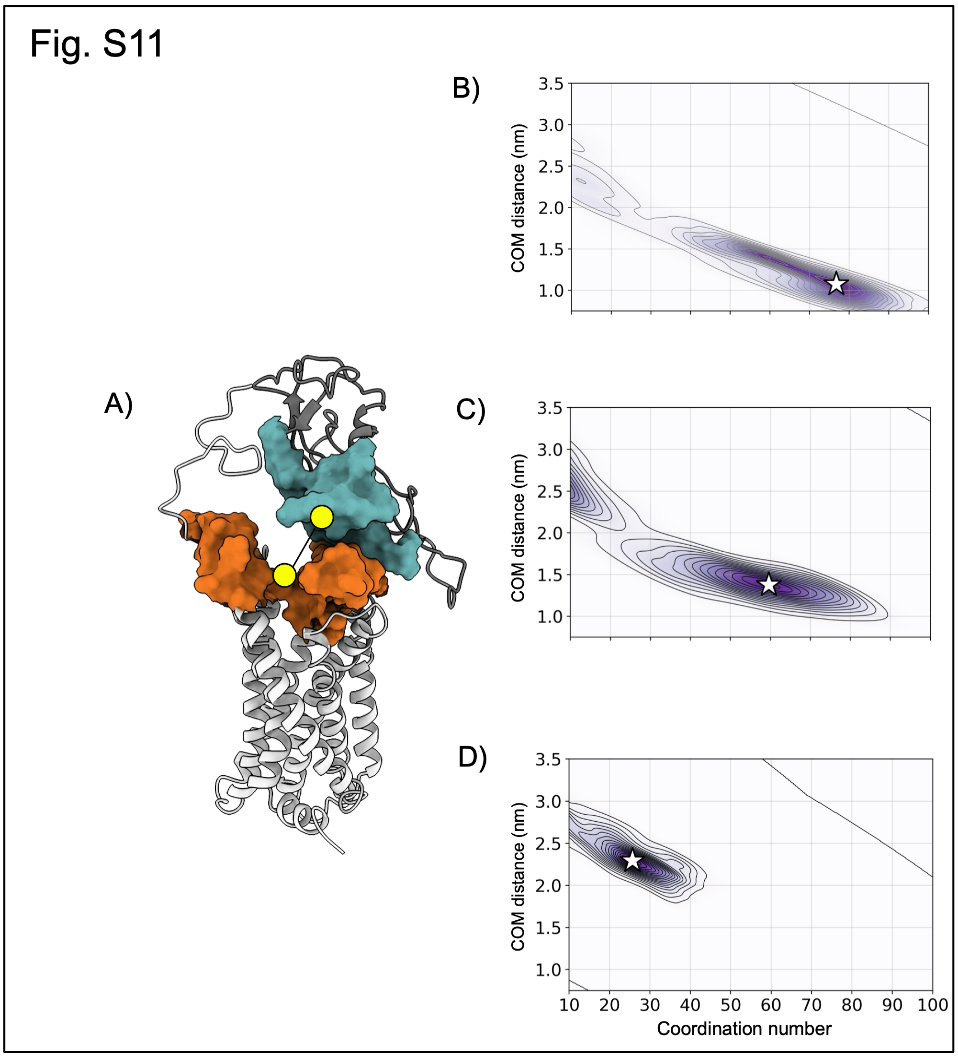

Figure S11. CloNe clustering of CCRL2–chemerin complexes (A) Structural depiction of clustering parameters: chemerin β1 and N-terminal (blue surface), CCRL2 ECL2 (orange). (B–D) Contour density plots for replicas 1–3 show conformation distribution by coordination and distance. The most populated cluster is marked by a white star.

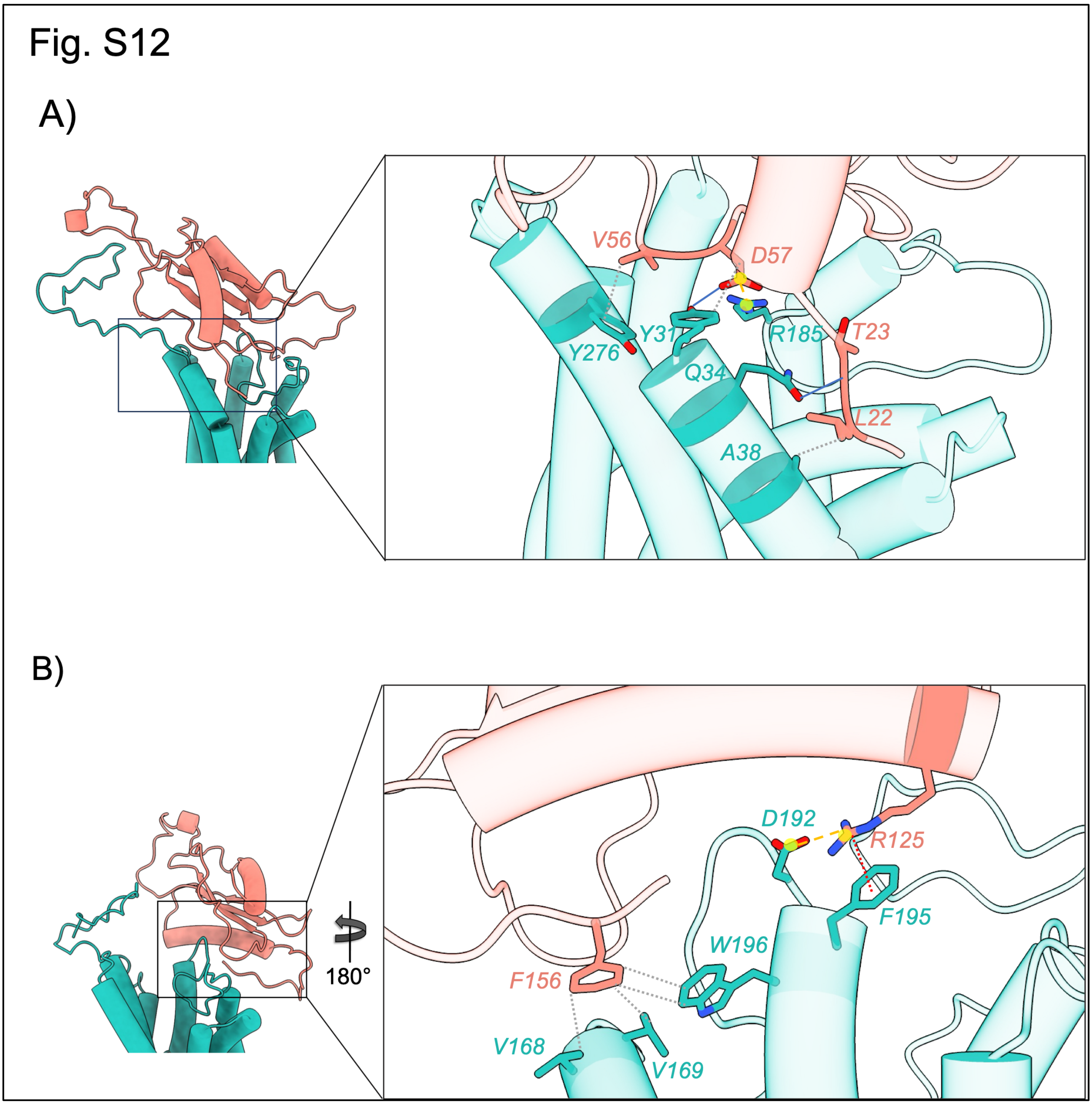

Figure S12. Interaction maps of CCRL2–chemerin complexes from dominant clusters. CCRL2 (light blue) and chemerin (light pink) interactions include hydrogen bonds (solid blue), hydrophobic contacts (dashed gray), and salt bridges (yellow dashed). (A) Replica 3 shows chemerin's α2 helix adopting an extended, solvent-shielded pose. (B) Loop2 of chemerin interacts with CCRL2's N-terminal domain.

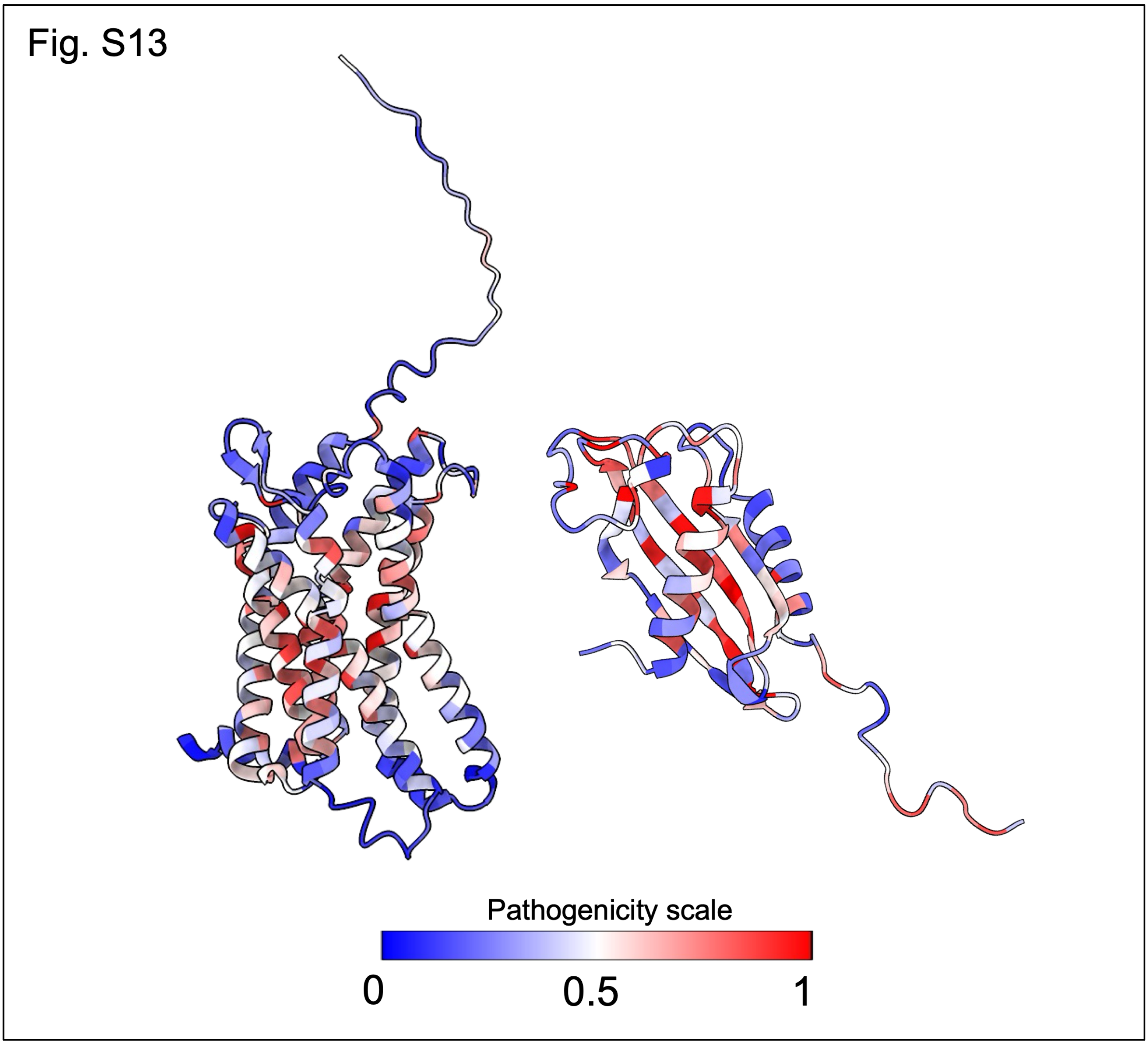

Figure S13. Structural mapping of CCRL2 and chemerin with AlphaMissense pathogenicity. Residues are colored by AlphaMissense pathogenicity scores: 0-0.33 (likely benign) in blue, 0.34- 0.564 (ambiguous) in white, and 0.565-1 (likely pathogenic) in red.

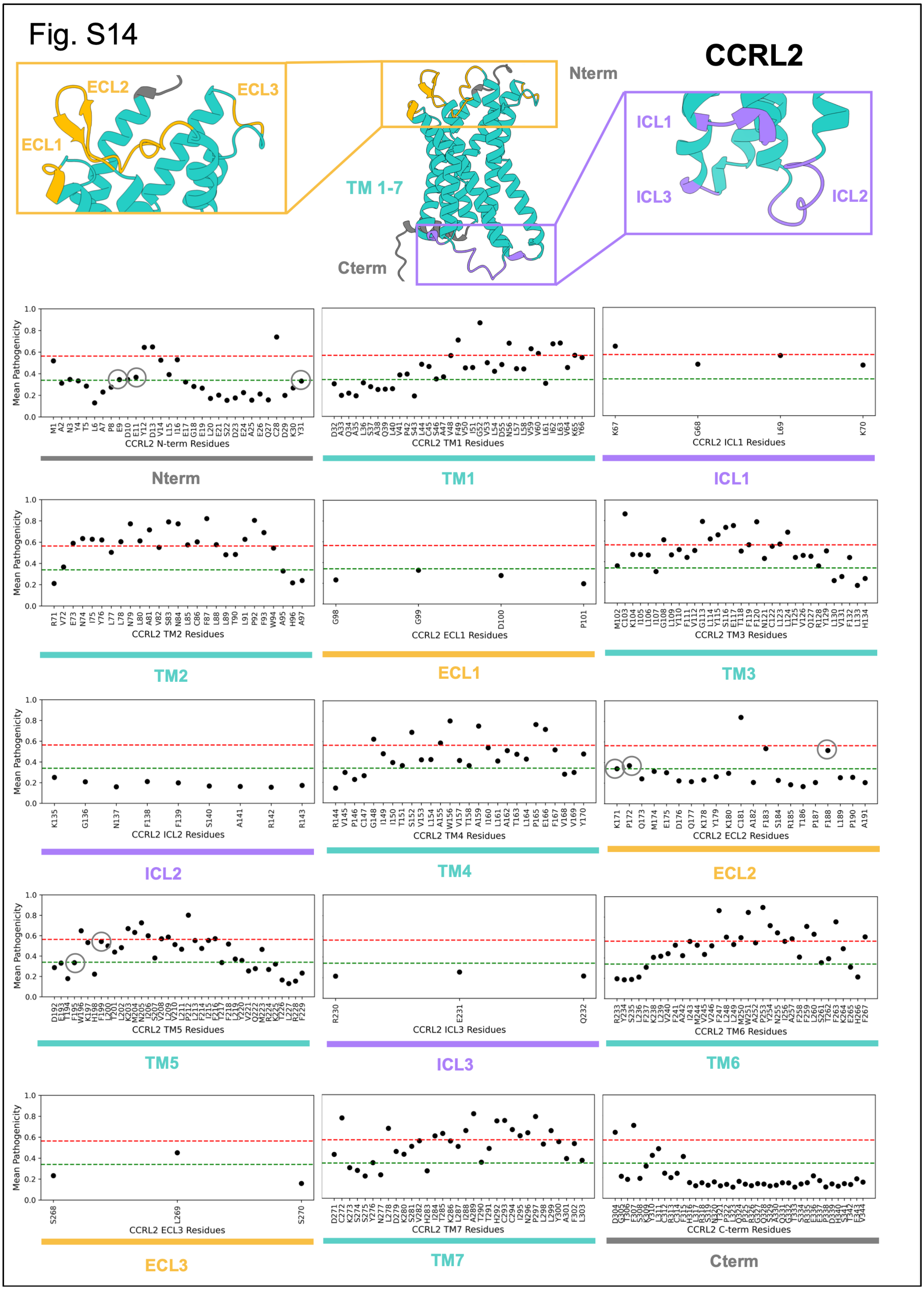

Figure S14. Regional pathogenicity mapping of CCRL2 residues. Dot plots show each residue's classification by AlphaMissense: above the red line (likely pathogenic), between red and green (ambiguous), below green (benign). Circled dots indicate residues with known interaction relevance.

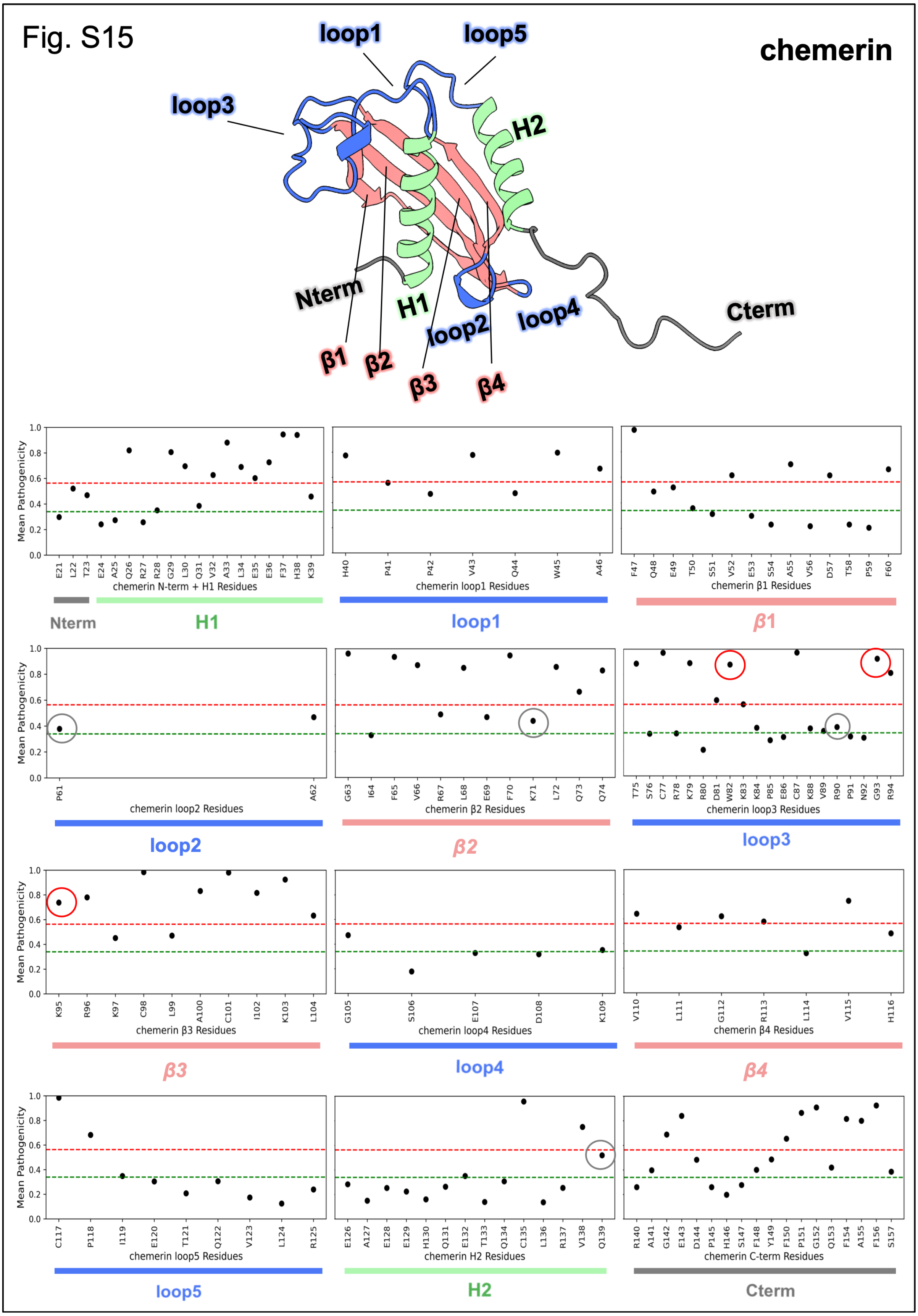

Figure S15. Pathogenicity mapping for chemerin (S13). Dot plots follow the same format as in S14, with interaction-relevant residues circled.

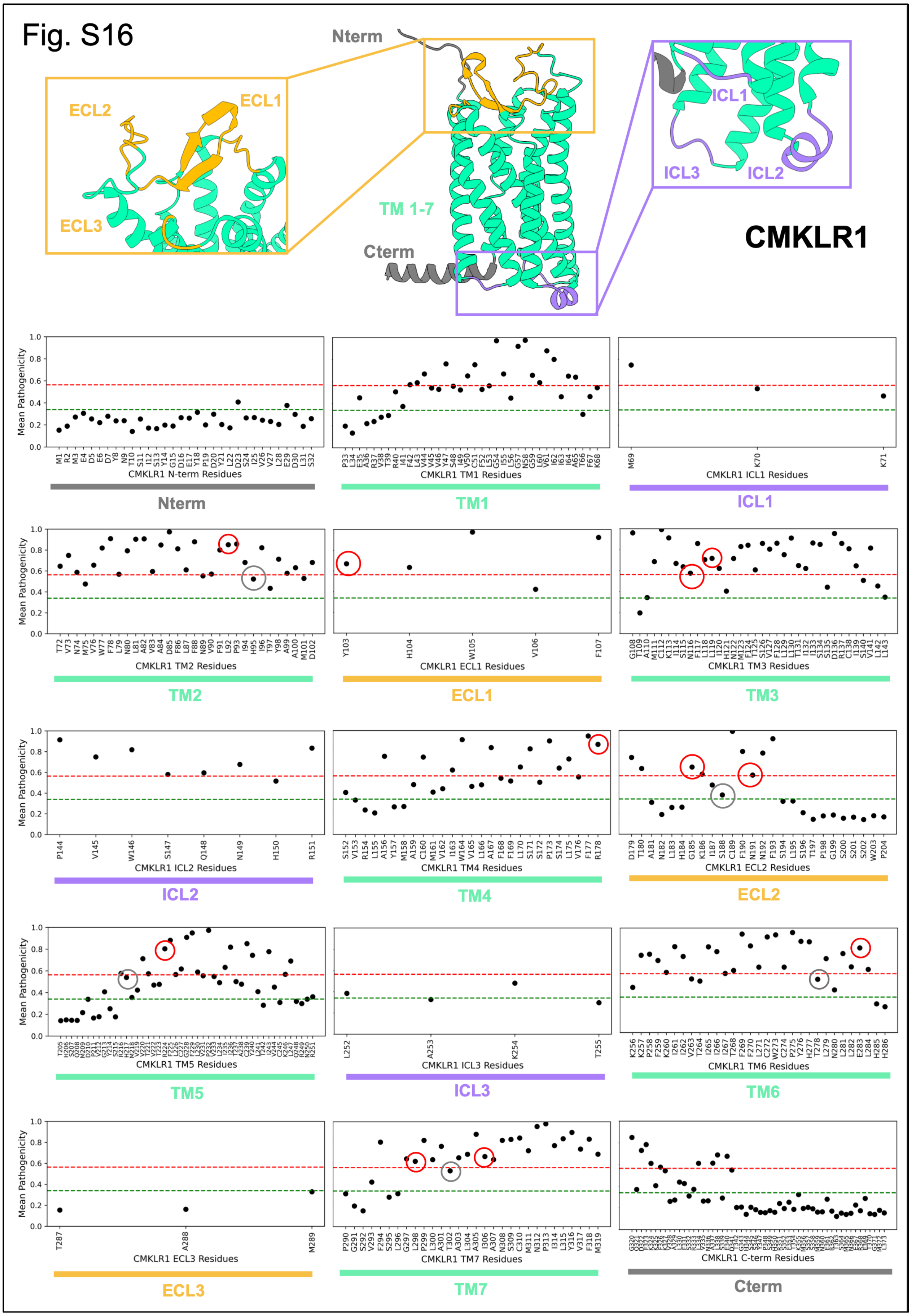

Figure S16. and CMKLR1 residues. Dot plots follow the same format as in S14, with interaction-relevant residues circled.

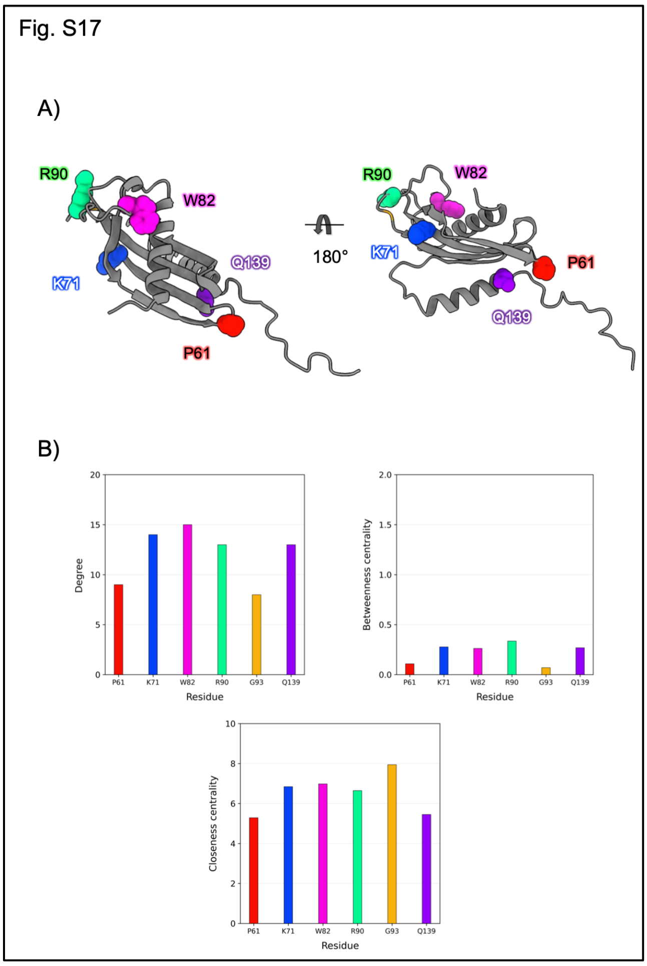

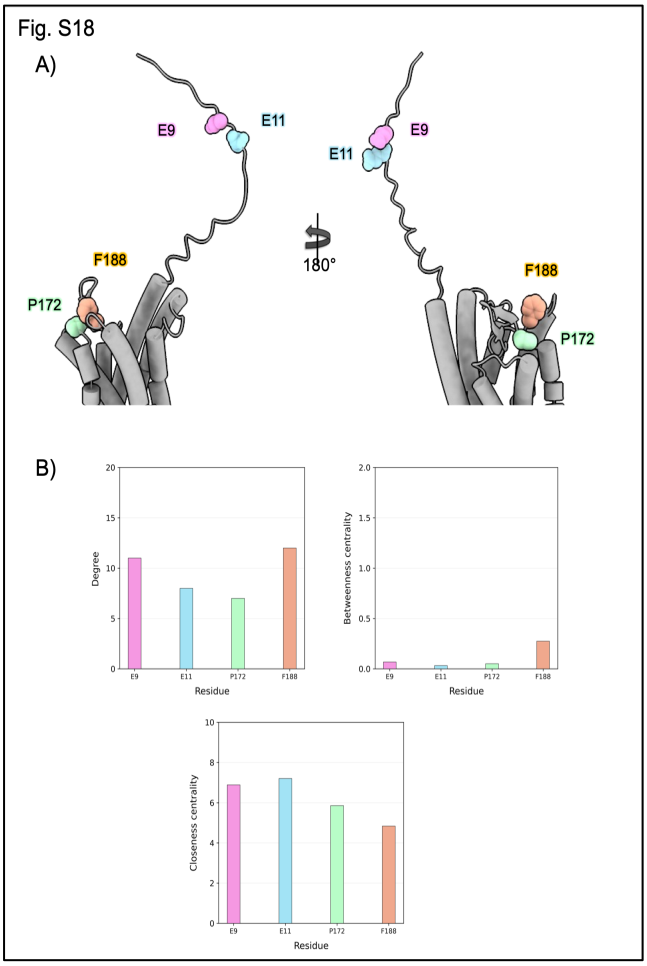

Figure S17–S18. Network analysis of chemerin (S17) and CCRL2 (S18) residues destabilizing the complex. (A) Key residues (G93, P61, R90, etc.) are shown as colored spheres on chemerin. (B) Bar plots depict Degree, Betweenness, and Closeness Centrality. Solvent-accessible surface area was zero for all residues.

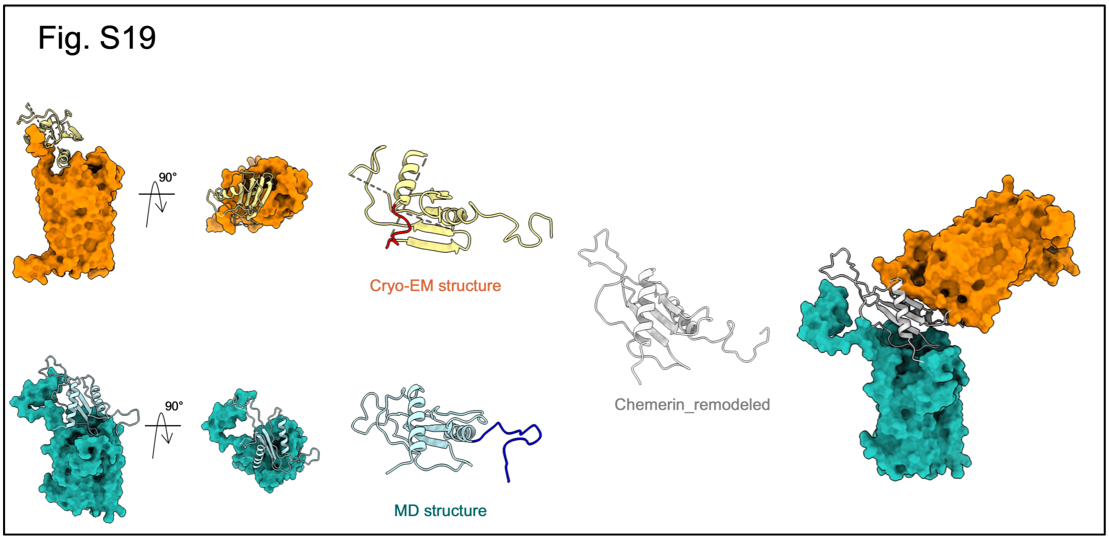

Figure S19. Final CCRL2–chemerin–CMKLR1 complex model integrating cryo-EM and MD data. The final model overlays the cryo-EM CMKLR1–chemerin (orange-yellow) with our CCRL2-chemerin model (blue-light blue). The cryo-EM data guided C-terminal orientation, while MD simulations informed N-terminal positioning and loop reconstruction.

Supplementary Tables

Table S1

Replica ID	Simulation time (μs)	Binding outcome	Extension strategy	Notes
R1	4.5	Stable binding	1.5 → 3.0 → 4.5	Extended due to stable contacts
R2	1.5	No binding	stopped at 1.5	Chemerin remained >30 Å
R3	1.5	No binding	stopped at 1.5	Chemerin remained >30 Å
R4	4.5	Stable binding	1.5 → 3.0 → 4.5	Extended due to stable contacts
R5	1.5	No binding	stopped at 1.5	Chemerin remained >30 Å
R6	4.5	Stable binding	1.5 → 3.0 → 4.5	Extended due to stable contacts
R7	4.5	Stable binding	1.5 → 3.0 → 4.5	Extended due to stable contacts
R8	1.5	No binding	stopped at 1.5	Chemerin remained >30 Å
R9	4.5	Stable binding	1.5 → 3.0 → 4.5	Extended due to stable contacts
R10	4.5	Stable binding	1.5 → 3.0 → 4.5	Extended due to stable contacts
R11	4.5	Stable binding	1.5 → 3.0 → 4.5	Extended due to stable contacts
R12	1.5	No binding	stopped at 1.5	Chemerin remained >30 Å
R13	4.5	Stable binding	1.5 → 3.0 → 4.5	Extended due to stable contacts
R14	3	Transient	1.5 → 3.0	Contacts but no stable interface
R15	3	Transient	1.5 → 3.0	Contacts but no stable interface
R16	4.5	Stable binding	1.5 → 3.0 → 4.5	Extended due to stable contacts
R17	1.5	No binding	stopped at 1.5	Chemerin remained >30 Å
R18	1.5	No binding	stopped at 1.5	Chemerin remained >30 Å
R19	1.5	No binding	stopped at 1.5	Chemerin remained >30 Å
R20	1.5	No binding	stopped at 1.5	Chemerin remained >30 Å
R21	1.5	No binding	stopped at 1.5	Chemerin remained >30 Å
R22	4.5	Stable binding	1.5 → 3.0 → 4.5	Extended due to stable contacts
R23	3	Transient	1.5 → 3.0	Contacts but no stable interface
R24	3	Transient	1.5 → 3.0	Contacts but no stable interface
R25	1.5	No binding	stopped at 1.5	Chemerin remained >30 Å
R26	4.5	Stable binding	1.5 → 3.0 → 4.5	Extended due to stable contacts

Table S1. Overview of the 26 coarse-grained CCRL2–chemerin simulations. All replicas (R1–R26) were run for 1.5 μs; unbound cases (>30 Å) were stopped, approaching cases were extended to 3 μs, and stable binding cases to 4.5 μs. The table lists simulation time, binding outcome, extension strategy, and key observations.

Table S2

CCRL2			
**gnomAD ID**	**Missense mutation**	**AlphaMissense result**	**Allele frequency**
3-46408106-G-T	p.E9D	Amb	6.28e-07
3-46408111-A-C	p.E11A	Amb	6.27e-06
3-46408111-A-G	p.E11G	Amb	3.13e-06
3-46408170-T-A	p.Y31N	Amb	6,20e-007
3-46408170-T-C	p.Y31H	Amb	1,24e-006
3-46408593-C-A	p.P172T	Amb	2.48e-06
3-46408594-C-T	p.P172L	Amb	6.2e-07
3-46408641-T-A	p.F188I	Amb	7.43e-06
**chemerin**			
**gnomAD ID**	**Missense mutation**	**AlphaMissense result**	**Allele frequency**
7-150340198-G-A	p.P61S	Amb	4.499645813593e-06
7-150340168-T-G	p.K71Q	Amb	6.429511974323e-07
7-150340166-C-G	p.K71N	Amb	6.429346624078e-07
7-150340135-A-C	p.W82G	Path	6.428065447991e-07
7-150340109-C-A	p.R90S	Amb	6.225471641731e-07
7-150340102-C-T	p.G93R	Path	1.23972116191e-06
7-150338702-G-C	p.Q139E	Amb	3.71719574752e-06

Table S2. Missense variant summary for CCRL2 and chemerin. Variants were extracted from gnomAD and filtered for predicted pathogenicity and interface relevance.

Table S3

CMKLR1			
**gnomAD ID**	**Missense mutation**	**AlphaMissense result**	**Allele frequency**
12-108292431-G-A	p.R178W	Path	6.1950806103889E-07
12-108292430-C-T	p.R178Q	Path	6.19510363788876E-07
12-108292391-T-A	p.N191I	Path	1.23910516781201E-06
12-108292391-T-C	p.N191S	Path	0.000001858696904774
**chemerin**			
**gnomAD ID**	**Missense mutation**	**AlphaMissense result**	**Allele frequency**
7-150338662-C-T	p.G152E	Path	6.23582130131613E-07

Table S3. Missense variant summary for CMKLR1, and chemerin. Variants were extracted from gnomAD and filtered for predicted pathogenicity and interface relevance.

Table S4

CCRL2						
	FoldX		MutaBind2		DynaMut2	DDMut-PPI
Missense mutation	ΔΔG protein stability (kcal mol^{-1})	ΔΔG protein interaction (kcal mol^{-1})	ΔΔG (kcal mol^{-1})	Deleterious?	ΔΔG protein stability (kcal mol^{-1})	ΔΔG protein interaction (kcal mol^{-1})
p.E9D	0.127	0.0051	1.71	yes	- 0.39	- 0.693
p.E11A	3.89	4.64713	2.51	yes	- 1.12	- 4.666
p.E11G	4.307	4.85384	2.56	yes	- 1.05	- 5.089
p.Y31N	1.387	1.647	0.99	no	1.18	- 1.041
p.Y31H	1.673	1.3765	0.68	no	0.96	- 0.562
p.P172T	9.099	4.55682	1.57	yes	- 0.64	- 1.456
p.P172L	2.301	0.88285	0.88	no	- 0.51	0.418
p.F188I	1.077	1.02194	1.11	no	- 1.67	- 0.187
**chemerin**						
	FoldX		MutaBind2		DynaMut2	DDMut-PPI
Missense mutation	ΔΔG protein stability (kcal mol^{-1})	ΔΔG protein interaction (kcal mol^{-1})	ΔΔG (kcal mol^{-1})	Deleterious?	ΔΔG protein stability (kcal mol^{-1})	ΔΔG protein interaction (kcal mol^{-1})
p.P61S	2.756	2.1723	2.53	yes	- 1.47	0.135
p.K71Q	- 0.57	- 0.257	0.82	no	- 1.33	- 0.177
p.K71N	- 0.688	- 0.3741	1.06	no	- 1.34	- 0.23
p.W82G	1.681	1.33534	1.75	yes	- 2.78	- 0.1432
p.R90S	3.464	4.71406	2.79	yes	- 1.71	- 0.583
p.G93R	3.503	- 0.342	2.43	yes	- 1.00	- 0.785
p.Q139E	- 0.013	- 0.4821	- 0.36	no	0.07	- 0.052

Table S4. ΔΔG-based mutation impact on CCRL2–chemerin binding stability. Stability predictions from FoldX, MutaBind2, DynaMut2, and DDMut-PPI. MutaBind2 classifies ΔΔG ≥ 1.5 kcal/mol as deleterious.

Table S5

CCRL2				
**Residue**	**Solvated area**	**Degree**	**Betweenness centrality**	**Closeness centrality**
E9	0	11	0.068	6.885
E11	0	8	0.032	7.203
P172	0	7	0.051	5.857
F188	0	12	0.274	4.835
**chemerin**				
**Residue**	**Solvated area**	**Degree**	**Betweenness centrality**	**Closeness centrality**
P61	0	9	0.108	5.289
K71	0	14	0.278	6.841
W82	0	15	0.262	6.98
R90	0	13	0.336	6.645
G93	0	8	0.07	7.943
Q139	0	13	0.269	5.452

Table S5. Protein Contacts Atlas analysis of CCRL2 and chemerin. Metrics include solvated surface area, degree, betweenness, and closeness centrality for destabilizing residues identified by FoldX.
